# Latent transcriptional variations of individual *Plasmodium falciparum* uncovered by single-cell RNA-seq and fluorescence imaging

**DOI:** 10.1371/journal.pgen.1008506

**Published:** 2019-12-19

**Authors:** Katelyn A. Walzer, Hélène Fradin, Liane Y. Emerson, David L. Corcoran, Jen-Tsan Chi

**Affiliations:** 1 Department of Molecular Genetics and Microbiology, Duke University, Durham, North Carolina, United States of America; 2 Center for Genomic and Computational Biology, Duke University, Durham, North Carolina, United States of America; University of Pennsylvania, UNITED STATES

## Abstract

Malaria parasites follow a complex life cycle that consists of multiple stages that span from the human host to the mosquito vector. Among the species causing malaria, *Plasmodium falciparum* is the most lethal, with clinical symptoms manifesting during the intraerythrocytic developmental cycle (IDC). During the IDC, *P*. *falciparum* progresses through a synchronous and continuous cascade of transcriptional programming previously established using population analyses. While individual parasites are known to exhibit transcriptional variations to evade the host immune system or commit to a sexual fate, such rare expression heterogeneity is largely undetectable on a population level. Therefore, we combined single-cell RNA-sequencing (scRNA-seq) on a microfluidic platform and fluorescence imaging to delineate the transcriptional variations among individual parasites during late asexual and sexual stages. The comparison between asexual and sexual parasites uncovered a set of previously undefined sex-specific genes. Asexual parasites were segregated into three distinct clusters based on the differential expression of genes encoding SERAs, rhoptry proteins, and EXP2 plus transporters. Multiple pseudotime analyses revealed that these stage-specific transitions are distinct. RNA fluorescent *in situ* hybridization of cluster-specific genes validated distinct stage-specific expression and transitions during the IDC and defined the highly variable transcriptional pattern of EXP2. Additionally, these analyses indicated huge variations in the stage-specific transcript levels among parasites. Overall, scRNA-seq and RNA-FISH of *P*. *falciparum* revealed distinct stage transitions and unexpected degrees of heterogeneity with potential impact on transcriptional regulation during the IDC and adaptive responses to the host.

## Author summary

Malaria poses a significant global health problem and is responsible for nearly 445,000 deaths. It is caused by a single-celled parasite that infects a human host and propagates asexually through red blood cells. The most deadly malaria parasite, *P*. *falciparum*, follows a tightly synchronous life cycle characterized by a continuous cascade of gene expression programming. Beyond these changes in the population, individual *P*. *falciparum* parasites are known to exhibit unique and rare expression, particularly to evade the host immune system and also to commit to a sexual fate. But such rare expression is often masked in bulk-cell studies. To understand expression variations among individual parasites, we combined single-cell RNA sequencing and fluorescence imaging to study the gene expression among individual asexual and sexual parasites. We discovered previously undefined sex-specific genes as well as three distinct clusters of late stage asexual parasites largely defined by stage-specific genes. We also showed that EXP2, a marker gene of one cluster, was expressed at multiple times throughout the life cycle. Furthermore, stage-specific gene expression was greatly variable among individual parasites. Our results have great implications for individual parasite survival and adaptation, particularly in response to environmental stresses like the host immune system.

## Introduction

Malaria persists as a global health problem, killing nearly 445,000 per year worldwide and resulting in 216 million new cases in 2016 [1]. *Plasmodium falciparum* is responsible for the majority of these deaths, making it the most virulent of the *Plasmodium* species. This unicellular parasite has a complex life cycle, including multiple stages in the human host and *Anopheles* mosquito vector. In particular, the parasite undergoes a 48-hour intraerythrocytic developmental cycle (IDC) in humans, with gene expression commonly thought to occur in a continuous cascade and only when it is needed in the asexual ring, trophozoite, and schizont stages [2]. To examine these distinct transcriptional changes, the parasite population was studied on a bulk-cell level after chemical synchronization [2–6]. In spite of synchronization, the *P*. *falciparum* population is known to be heterogeneous. For example, one to thirty percent of parasites commits to a sexual fate [7–9] and develops into male and female gametocytes, the only stage of the parasite transmissible to the mosquito. Parasites within the same population may also exhibit known and unknown clonal variations, as shown in the parasites’ antigenic variations to evade the host immune system [10]. This suggests that transcript expression is variable from cell-to-cell, an occurrence that is missed when averaged in bulk-cell analyses, even with chemical synchronization.

To overcome the issue of complexity in cellular populations, many groups have applied single-cell RNA-sequencing (scRNA-seq) to uncover cellular heterogeneity and rare gene expression. These findings include the discovery of rare intestinal cell types [11], bimodal gene expression and splicing in immune cells [12], and regulatory variation before and after stress in budding yeast [13]. Recently, scRNA-seq of *P*. *falciparum* uncovered genes upregulated in sexually committed cells [14, 15], genes upregulated in early stage gametocytes [16], discrete transcriptional changes during the asexual cycle [17], and genes expressed during the entire life cycle, including mosquito stages [18]. These approaches varied in their capture of the single-parasite transcriptome, from thousands of cells with low-coverage (Drop-seq and 10x Genomics) [14, 18–20] to hundreds of cells with either low-coverage (SCRB-seq) [15, 21] or full-length transcript coverage (Smart-Seq2) [16–18, 22]. By capturing thousands of synchronized asexual cells at three time points, scRNA-seq with Drop-seq resolved cell cycle progression of individual parasites into 11 clusters [14]. Higher coverage Smart-Seq2 also organized single-parasite transcriptomes by pseudotime but relied on previously published bulk-cell analyses to verify cell cycle progression without independent validation, potentially missing rare and uncharacterized gene expression [17].

Here, we describe our development of a single-cell workflow to uncover the heterogeneity of *P*. *falciparum* populations using the Fluidigm C1 for scRNA-seq, followed by validation with RNA fluorescent *in situ* hybridization (RNA-FISH). To establish our single-cell methods, we first compared expression of single asexual parasites and late-stage gametocytes, uncovering a large number of previously undefined gametocyte-specific genes. We were then able to separate individual trophozoites and schizonts into three distinct clusters based on their specific gene expression. These three clusters were characterized by the expression of exported proteins and transporters, SERA genes, and rhoptries, respectively. This was further verified using RNA-FISH with markers for each cluster: EXP2, SERA4, and RAP1. While two clusters (SERA and rhoptry genes) were verified to be cell-cycle dependent, a gene in the third cluster, EXP2, showed expression throughout multiple times of the life cycle, particularly at the early trophozoite and late schizont stages. Additionally, we have found that expression of the stage-specific marker genes is highly variable among individual parasites and that transition between stages is shorter than and not as smooth as previously thought. Overall, these data allow us to overcome the limitation of bulk-cell analysis and decipher which genes are expressed in each individual cell. Our study highlights the power that single-cell analyses have with regards to dissecting complex populations of cells to uncover hidden gene expression patterns in *P*. *falciparum*. We expect that a similar approach can be applied to other eukaryotic pathogens to enhance our understanding of cell-to-cell heterogeneity and the functional consequences of such variability.

## Results

### Development of single-cell RNA-seq for *P*. *falciparum*

Studies on *P*. *falciparum*, including large-scale transcriptomic and proteomic profiling of multiple stages of the life cycle [2–6, 23], have mostly focused on bulk-population analyses of synchronized parasites. Even with chemical synchronization, it is possible that there is still significant heterogeneity or rare populations that are masked by bulk-cell analyses that only measure the average gene expression across all cells. Thus, only single-cell studies can reveal previously unrecognized cellular heterogeneity and potential rare/novel cell types. To this end, we used the microfluidic technology of the Fluidigm C1 to capture single parasites and perform scRNA-seq with full transcript coverage. We captured and analyzed 46 asexual parasites and 5 late-stage gametocytes to compare between asexual- and sexual-stage parasites as well as identified the subtypes among asexual parasites. The results from scRNA-seq were further validated using RT-PCR, RNA-FISH, and previously published scRNA-seq data [17].

**Fig 1:**
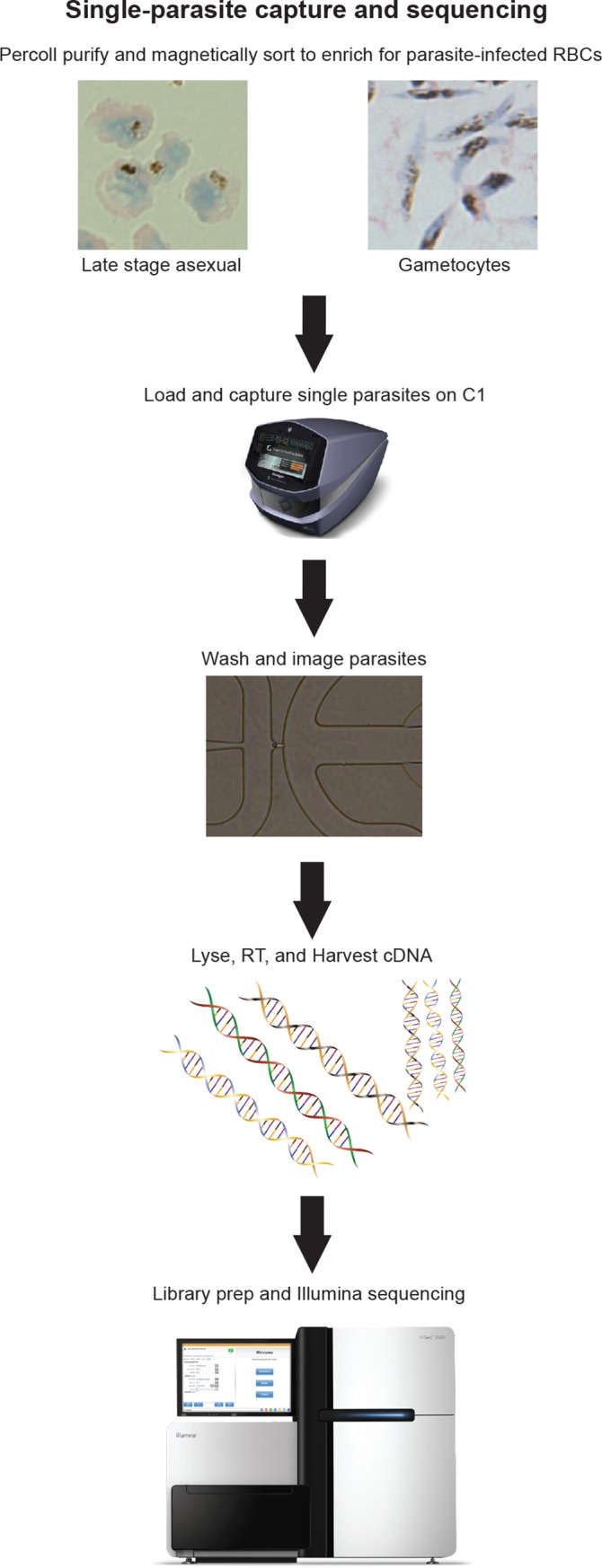
The workflow of parasite isolation for scRNA-seq. Late stage asexual parasites were collected 34 hours post-invasion while stage IV-V gametocytes were collected on day 9. Either trophozoites and schizonts or late stage gametocytes were first separated from rings, early gametocytes, and uninfected RBCs by a 40/70% Percoll density gradient and were further purified from uninfected RBCs by MACS magnetic sorting. These infected RBCs were loaded onto a C1 IFC for single-cell capture. Each well was visually inspected to ensure the presence of a single parasite. Then the single parasites were lysed and cDNA was synthesized using SMARTer-Seq chemistry. The harvested cDNA was then used to generate a sequencing library for Illumina sequencing.

**Fig 2:**
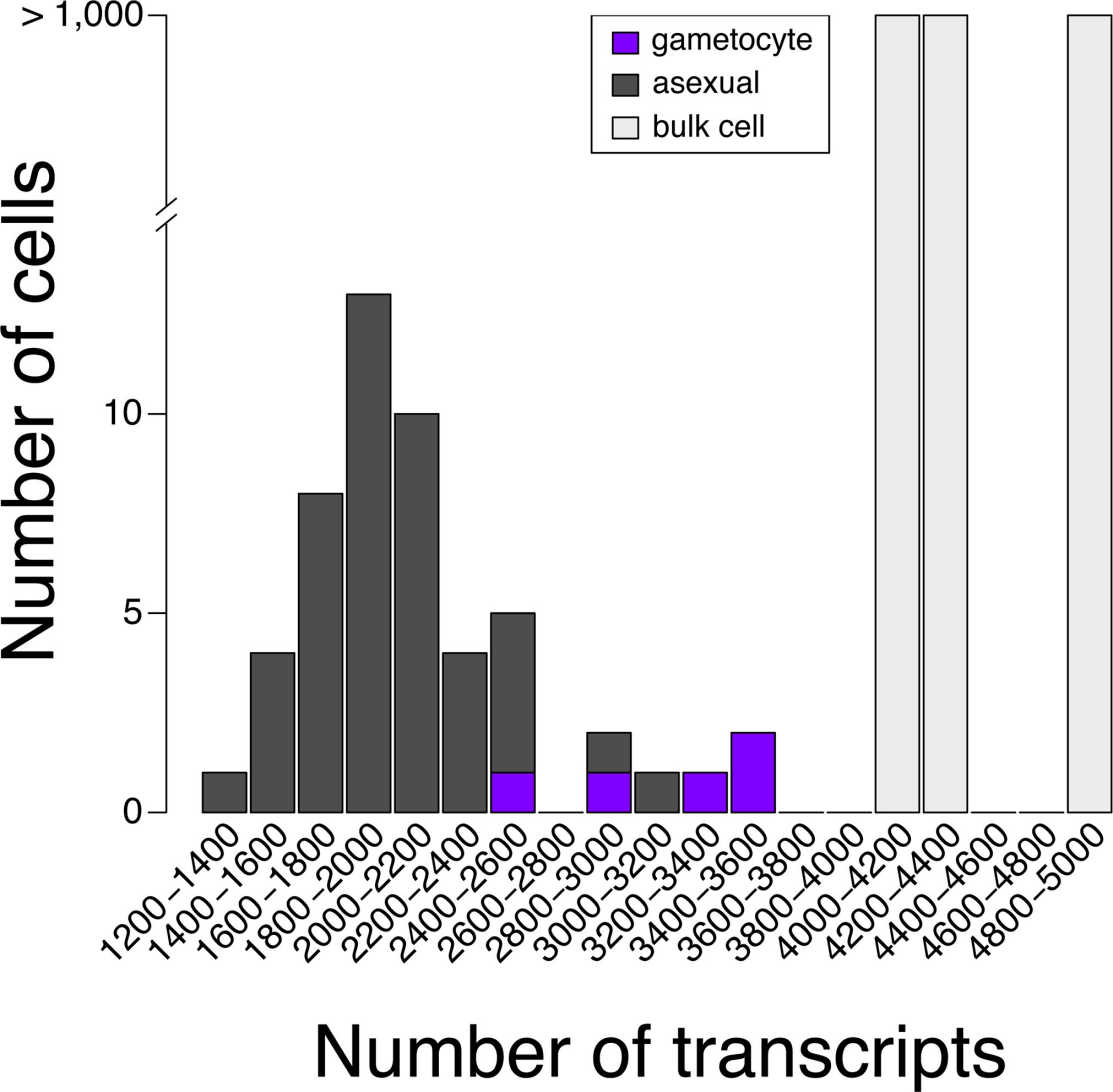
The distribution of expressed transcripts in individual asexual cells, gametocytes, or the total parasite population. Generally, single gametocytes expressed more genes than single asexual cells.

Parasite cultures were sorbitol synchronized twice, each 48 hours apart, before collection of late stage asexual parasites that were approximately 34 hours post-invasion. Gametocytes were enriched in synchronized cultures by treatment with 50 mM N-acetyl glucosamine for 72 hours and were collected 9 days (stage IV-V) after drug treatment began. Parasite-infected erythrocytes were purified from uninfected erythrocytes by a 40/70% Percoll density gradient and MACS before being loaded onto the Fluidigm C1 Integrated Fluidic Circuit (IFC) for single-cell capture (Fig 1). Each microfluidic chamber was visually inspected under an inverted microscope to validate the presence of a single parasite before further analysis. While late-stage gametocytes had high capture rates (77 percent and above) on the IFC, trophozoite and schizont capture proved more challenging. We tested various ratios of cell number to suspension buffer using a concentration of 400 cells/μL and found that 40 to 50 percent of cells making up the final suspension buffer was optimal, although capture rates were still low (between 24 to 27 percent upon visual inspection). Therefore, we used two IFCs to capture a total of 46 single asexual parasites for scRNA-seq library construction using a Smart-Seq protocol. Sequencing was performed on an Illumina HiSeq with single-end 50 bp reads. Generally, gametocytes expressed a greater number of transcripts than asexual cells (Fig 2). On a bulk-cell level, 4805 transcripts were detected in late-stage gametocytes while 4136 and 4313 transcripts were detected in two different asexual samples. At a single-cell level, this number was much lower in both cell types with an average of 3127 transcripts detected in gametocytes. In individual asexual cells, 1321 to 3071 transcripts, with an average of 2002 transcripts, were detected. This number of detected transcripts in our study is consistent with the average of 1712 detected transcripts found in previous scRNA-seq data [17]. Additionally, we analyzed the length distribution of all genes and expressed genes and noted a slight bias towards longer transcripts (S1 Fig), indicating that both long and short transcripts were well represented in the dataset.

### Single-cell RNA-seq identifies gametocyte-specific genes and separates asexual parasites into three distinct clusters

**Fig 3:**
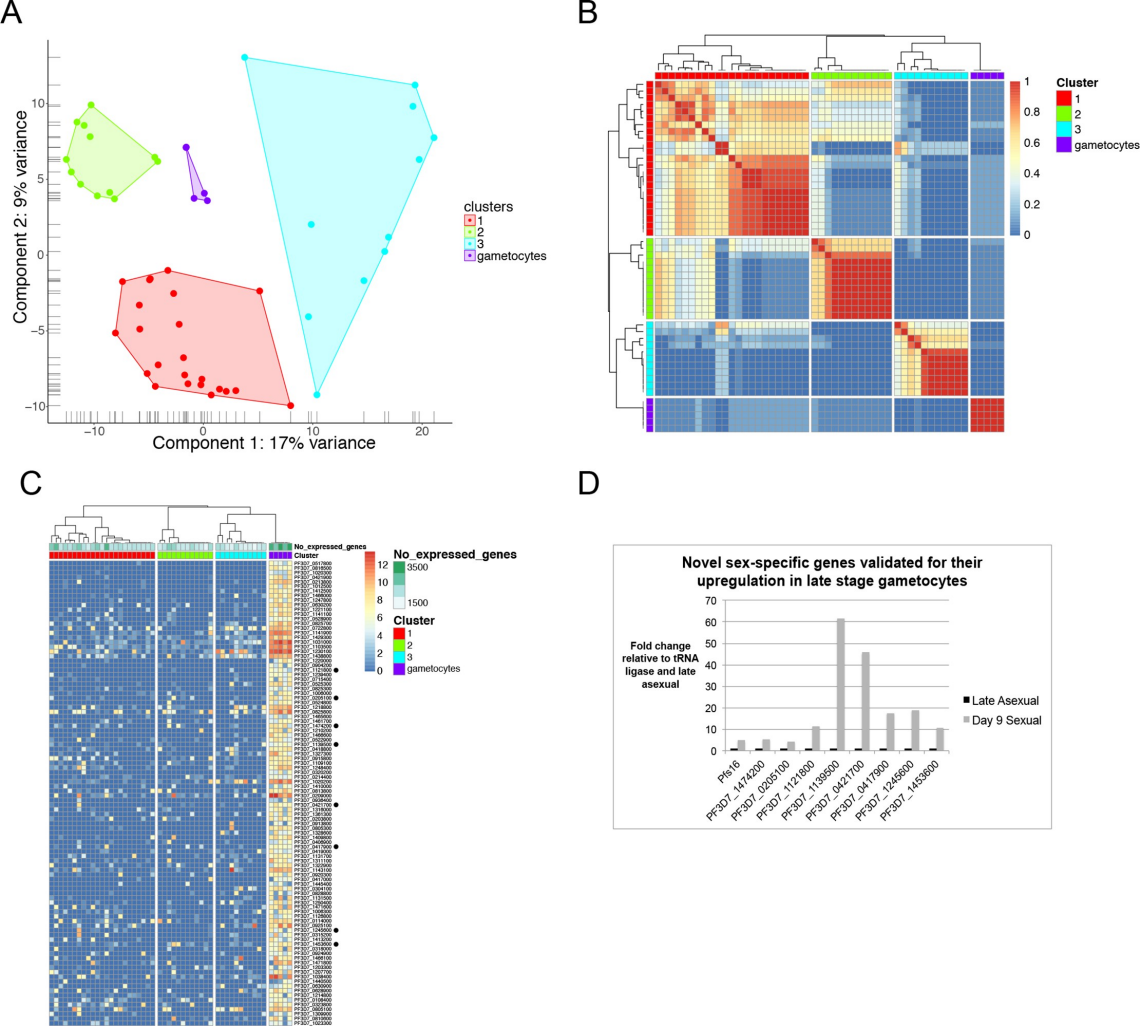
Single gametocytes cluster separately from three distinct asexual populations and express gametocyte-specific genes revealed by scRNA-seq. A) Principal component analysis (PCA) was used to visualize the clustering of 46 late stage asexual cells and 5 late stage gametocytes by gene expression highlighting clear separation of the gametocyte single cells from three distinct asexual populations. Two gametocytes had very similar first and second component coordinates, overlapping on the PCA plot. B) Consensus clustering matrix of all cell pairs displays very strong cell-to-cell similarity amongst gametocytes when compared to asexual cells, which are grouped together based on their similar gene expression. The color scale indicates the likelihood that two cells are arranged in the same cluster (blue: low (0), red: high (1)). C) 340 genes were specifically expressed in gametocytes relative to late stage asexual parasites, with the top 100 genes shown in this heatmap. The SC3 markers function was used to determine the potential that each gene could be a marker for the cluster in which its mean expression value was the highest. Genes are ordered by decreasing area under the receiver operating characteristic (AUROC) with a cutoff of 0.7. D) Eight novel gametocyte genes, denoted by black dots in (C), were chosen for qPCR validation on a bulk-cell level, n = 2. Gametocytes collected on day 9 were compared to late stage asexual parasites. Fold changes are relative to the tRNA ligase control and asexual samples. Pfs16 is a positive control for gametocyte-specific expression.

As a first step to analyze the heterogeneity of transcriptomes of the 51 single parasites, we performed unsupervised clustering using the clustering method implemented in SC3 [24]. A total of four stable clusters emerged: three clusters of asexual cells and one cluster of gametocytes, visualized using principal component analysis (PCA) (Fig 3A). Among the asexual clusters, cluster 1 was made up of 23 cells, cluster 2 had 12 cells, and cluster 3 had 11 cells. To examine the cell-to-cell similarities and differences within and between clusters, consensus clustering was performed and showed that cells in each cluster exhibited stronger similarity with each other than with cells in other clusters (Fig 3B). In particular, the 5 gametocytes showed very strong correlation with one another but little-to-no correlation with any asexual cells, indicating their distinct transcriptional profiles. Additionally, gametocytes are larger and generally express more transcripts, which may have contributed to their distinct clustering pattern away from asexual parasites.

Cluster-specific genes for gametocytes were identified using the R package SC3 [24]: 340 genes were upregulated in the gametocytes relative to the asexual cells, with an area under the receiver operating characteristic (AUROC) cutoff value of 0.7 (S5 Table). These markers were ordered according to their decreasing AUROC value, with the top 100 differentially expressed in gametocytes shown in Fig 3C. Some of the top expressed genes include known gametocyte markers P25 and P230 [25–27]. Among the 340 gametocyte genes, 185 are annotated as differentially transcribed in females and 73 are annotated as differentially transcribed in males [28]. From the top 100 genes, 8 gametocyte-expressed genes not previously described as gametocyte-specific along with the known marker Pfs16 were chosen for further qPCR validation using population samples from asexual parasites and gametocytes (Fig 3D). Four of these genes have unknown functions while the others putatively encode a peptidase, kinesin, RAP protein, and AAA family ATPase. These 8 genes were all shown to be upregulated in late-stage gametocytes when compared to the asexual stage, with six genes being upregulated more than 10-fold (Fig 3D). Over half of the 340 gametocyte markers have unknown functions, and future studies will prioritize these genes to understand their functional roles in gametocytogenesis and gametogenesis.

**Fig 4:**
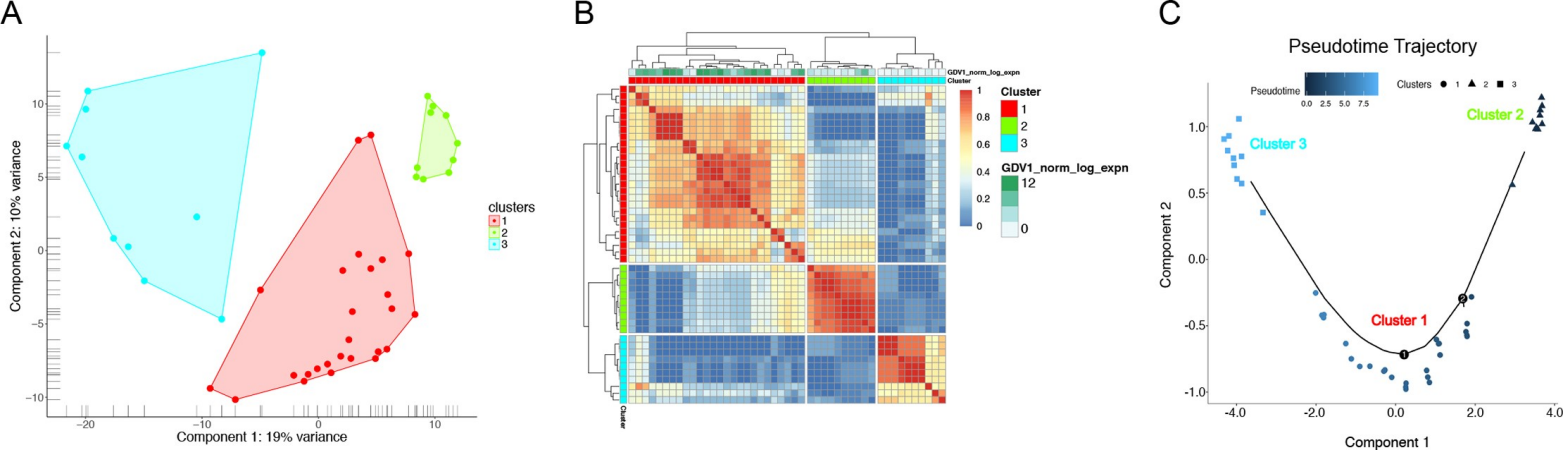
Single-cell RNA-seq expression of late stage asexual parasites identifies three distinct populations. A) Principal component analysis (PCA) was used to visualize the clustering of 46 single late stage asexual transcriptomes. B) Consensus clustering indicates that parasites within the same cluster show the strongest similarity when compared to parasites from other clusters. However, there is significant heterogeneity even between parasites in the same cluster. Blue indicates low similarity between two cells, whereas red indicates high similarity, i.e. that the two cells are always assigned to the same cluster. Single parasites are also annotated for their expression of GDV1 by its log normalized expression. C) Pseudotemporal ordering of 46 single asexual parasites by *monocle* 2. Genes expressed differentially between the 3 clusters detected by *SC3* were used for ordering cells and inferring a trajectory. Cells are colored by pseudotime and shapes indicate their assignment to one of the 3 clusters.

Having established that scRNA-seq clearly identifies distinct transcriptional differences between asexual parasites and gametocytes, we further evaluated the heterogeneity among the asexual population. After removing gametocytes, we performed unsupervised clustering using the clustering method implemented in SC3 [24], which separated the remaining 46 single asexual parasites into three distinct groups (Fig 4A). Cluster 1 was made up of 26 cells, cluster 2 had 10 cells, and cluster 3 had 10 cells. Since these individual asexual parasites were collected at the same time after synchronization, we performed consensus clustering to group the parasites by gene expression and define their similarities and differences across different clusters. Consensus clustering again indicated stronger similarity amongst cells within the same clusters than between them, although cell-to-cell heterogeneity within clusters is more evident after the removal of gametocytes, with some sub-clusters, especially in cluster 1 (Fig 4B), suggesting further heterogeneity within this cluster. In particular, the expression of GDV1, a gene involved in sexual commitment [9, 29], varied in cluster 1 with some high expessers and low expressers (Fig 4B). This indicates that gene expression for sexual commitment may contribute to the heterogeneity within the larger cluster 1. Additionally, multiple pseudotime analyses, with and without these clusters as an indicator variable, revealed the clear ordering of single parasites into three distinct and separate clusters, which started with cluster 2 and progressed to cluster 1, followed by cluster 3 (Fig 4C and S2 Fig).

**Fig 5:**
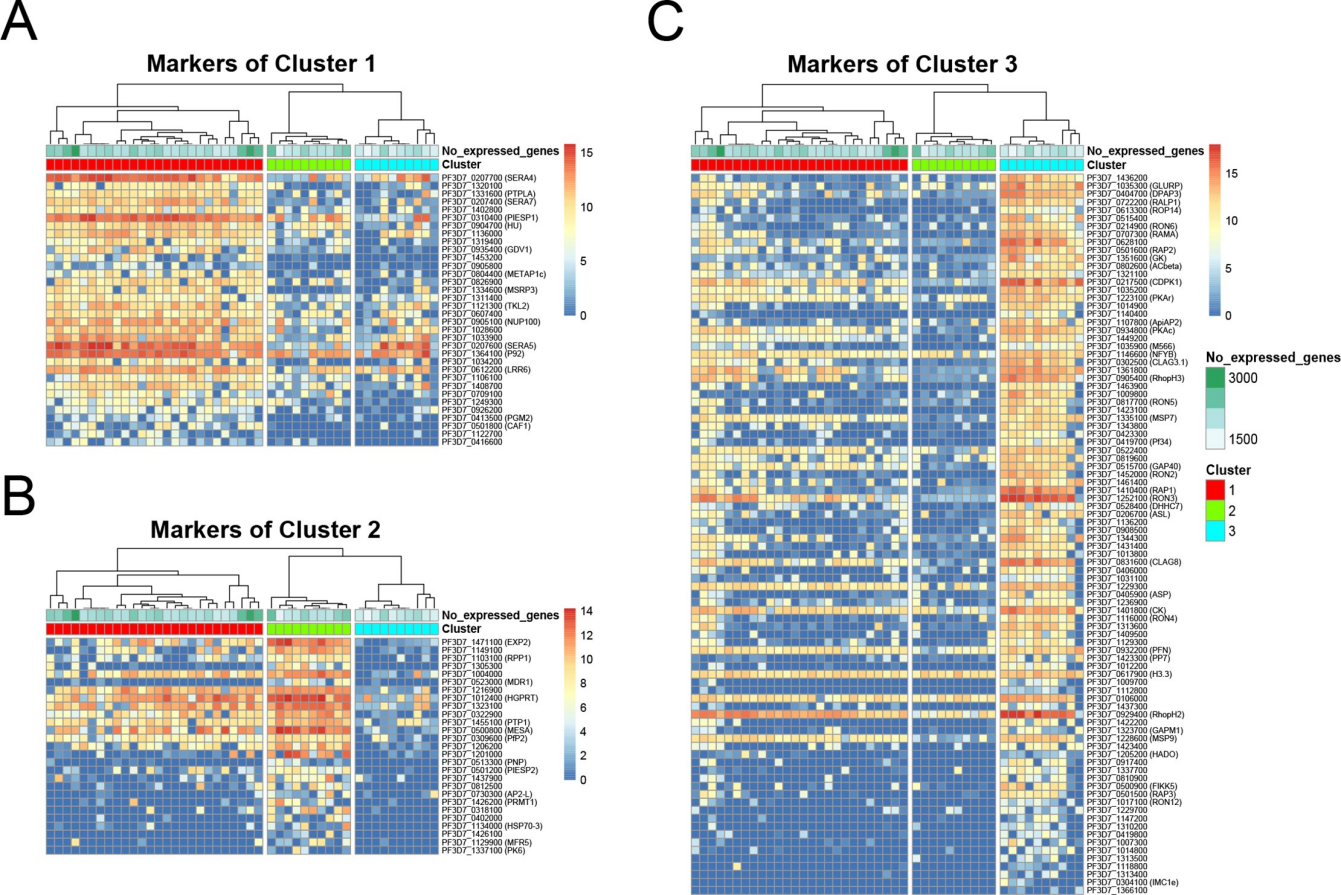
The top marker genes of each cluster were identified by scRNA-seq. The SC3 markers function was used to determine the potential that each gene could be a marker for the cluster in which its mean expression value was the highest. Genes are ordered by decreasing area under the receiver operating characteristic (AUROC) in clusters 1 (A), 2 (B), and 3 (C).

To identify the genes that best represent each cluster, we used the marker genes function of R package SC3 [24] and ranked the cluster-specific marker genes by AUROC values (Fig 5). Cluster 1 showed expression of many serine repeat antigen (SERA) genes, including SERA4, SERA5, and SERA7, as well as expression of GDV1 (Fig 5A). Cluster 2 was characterized by the expression of a number of exported proteins and transporters (Fig 5B). This includes EXP2, which is a member of the PTEX complex involved in the export of parasite proteins into the host erythrocyte [30, 31]. Recently, EXP2 was also characterized for its role in nutrient transport [32, 33]. MDR1 is another cluster 2 transporter and is found in the food vacuole; mutations in this gene are associated with chloroquine resistance [34]. Meanwhile, MFR5, another cluster 2 gene, was previously identified as a putative transport protein of the major facilitator superfamily (MFS) [35] and may transport similar solutes as other cluster 2 transporters. Known exported proteins among cluster 2 genes included MESA [36, 37], PIESP2 [38], and a PHIST domain-containing gene (PF3D7_0402000) [39], all important for remodelling the human erythrocyte during infection. Cluster 3 contained several rhoptry genes, including RAP1, ROP14, RON6, RAMA, RAP2, RhopH3, RON5, RON2, and RON3 (Fig 5C). Rhoptries are secreted proteins that function in the invasion pathway [40]. An essential AP2 factor (PF3D7_1107800) was also associated with this cluster, similarly to the rhoptry cluster in another scRNA-seq dataset [17]. This co-expression of AP2 and rhoptry genes may suggest a potential regulatory relationship that will need to be further investigated. Furthermore, the genes expressed in these clusters are consistent with our non-cluster indicator variable pseudotime analysis, which also exhibits two groups for cluster 1 (S3 Fig).

To validate our clusters and markers, we also analyzed another scRNA-seq dataset [17]. We processed the transcriptomes of 172 late-stage asexual *P*. *falciparum* the same way as our transcriptomes and clustered the parasites according to our 151 cluster-specific markers. We found that all of our identified markers were also expressed in these parasites (S6 Table) and that the optimal number of clusters was also three (S4 Fig). The 85 markers of the Reid et al. cluster 3 were all markers of our cluster 3. The Reid et al. cluster 2 shared 9 markers with our cluster 1 and 2 with our cluster 3, while the Reid et al. cluster 1 shared 24 markers with our cluster 2 but also 13 with our cluster 1. Of note, EXP2, MDR1, MFR5, MESA, PIESP2, and PF3D7_0402000 are all shared between Reid cluster 1 and our cluster 2 while all three SERA genes are shared between Reid cluster 2 and our cluster 1. GDV1 changed clusters and appears in Reid cluster 1 with the exported proteins, potentially indicating differences between sexual commitment rates or timing, which may be related to experimental conditions or use of a different parasite strain. Therefore, we could cross-validate our results using an independent scRNA-seq study [17].

When comparing the genes expressed in each group of our dataset, we noticed that genes in clusters 1 and 3 seemed to represent known stage-specific markers in the *P*. *falciparum* IDC. To validate these cluster-specific markers and determine their time-specific progression during the IDC, we employed two validation approaches, 1) comparison to previously published data of IDC progression and 2) RNA fluorescent *in situ* hybridization (RNA-FISH). First, we compared the cluster-specific genes to a previously published microarray dataset, which represents the most meticulous temporal analysis of the *P*. *falciparum* IDC to date [2]. As expected, genes in clusters 1 and 3 followed a largely time-specific pattern, with cluster 1 characteristic of a trophozoite-to-schizont transition and cluster 3 representative of schizonts (S5 Fig). As cells were synchronized during the ring stage a few hours after re-invasion, the majority of collected asexual single cells were predicted to be 34 hours post invasion (hpi). However, given the limitation of current synchronization methods, the exact timing of any individual parasite may vary. Cluster 1 had the most cells and exhibited strong expression of SERA4, SERA5, and SERA7 (Fig 5A). These genes are highly expressed at 34 hpi, confirming our capture of this stage of the parasite and their identity as cluster 1 markers (S5A Fig). Cluster 3 genes were also characteristic of cell cycle progression and represent schizonts past 34 hpi. In previous data, strong upregulation of cluster 3 genes begins at 32 hpi and continues through 48 hpi, confirming our cluster 3 and capture of these later-stage parasites (S5C Fig). Importantly, our single-cell data indicate that these genes are preferentially expressed in distinct groups of cells (Fig 5). Unlike the synchronized parasite population at 34 hpi which appears to express both SERA and rhoptry genes highly (S5 Fig), our single-cell transcriptomes show distinct transitions in gene expression in single parasites (Figs 4C and 5), a finding further validated using RNA-FISH.

In contrast, the genes denoted as markers of cluster 2 were not expressed at one particular stage in bulk-cell microarray data (S5B Fig). While some genes (e.g., MESA and PIESP2) are highly expressed in early trophozoites, other genes, including MDR1 and AP2-L, are expressed in late schizonts and early rings (S5B Fig). The expression timing of these cluster 2 markers is also distinct from the expected 34 hpi when we captured the parasites. However, because these genes cluster together in single-cell analysis, it appears that cluster 2 specific genes exhibit co-expression not seen previously on a bulk-cell level. In particular, EXP2, the top marker for cluster 2, is a component of the PTEX complex that is responsible for the export of *P*. *falciparum* proteins into host red blood cells. Because it shows co-expression with MDR1 and other transporter genes, EXP2 was chosen for further validation of its single-cell expression at multiple time points.

### RNA fluorescent *in situ* hybridization reveals distinct stage transitions and large variations in gene expression levels

To more clearly define the distinct cell types associated with expression heterogeneity, we used RNA-FISH as a second validation method to determine the expression levels of these cluster-specific transcripts over multiple time points of the IDC. Specifically, we chose SERA4, EXP2, and RAP1 as representative markers for each cluster. Parasites were collected approximately 32 to 38 hours post-synchronization and then staged based on size and morphology [41]. These cells were stained by RNA-FISH for the three markers simultaneously and scored for their stage and expression of each marker. In total, 647 parasites were counted, and 339 parasites exhibited expression of at least one of the three markers and were included in further analyses.

**Fig 6:**
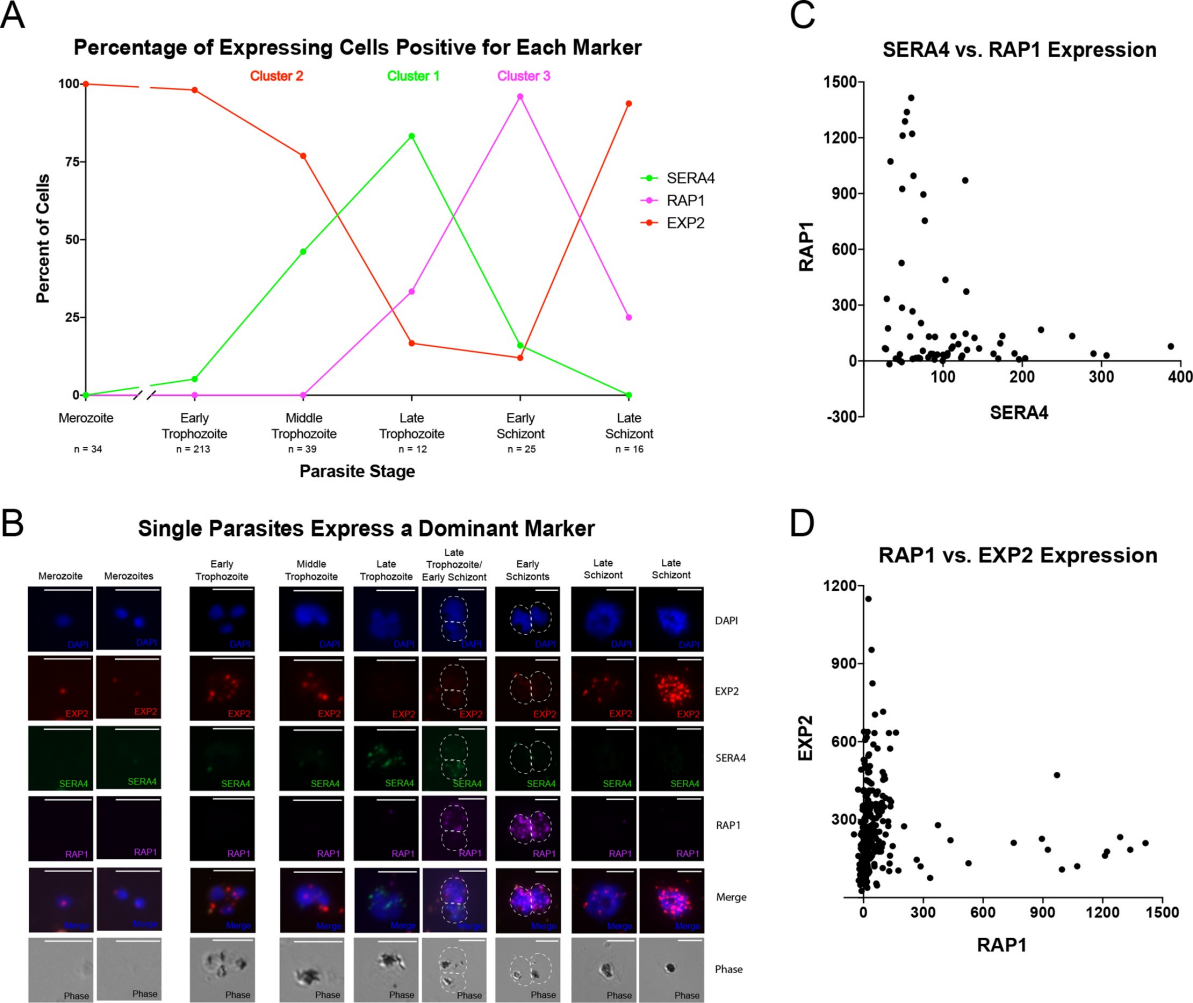
RNA-FISH reveals cell cycle progression of gene expression and distinct pattern among expressing cells for markers SERA4 (cluster 1), EXP2 (cluster 2), and RAP1 (cluster 3). A) The percentage of cells expressing each marker gene was determined for the morphological stage of the parasite and plotted over time, indicating distinct cell cycle progression of gene expression with some overlap between markers during stage transition. The number of expressing cells per stage is indicated by n. B) Representative images for expression of markers during cell cycle progression, with parasites expressing EXP2 (red), SERA4 (green), and RAP1 (magenta). Scale bar: 5 μm. C) The pair-wise correlation between expression values measured for RNA-FISH markers SERA4 and RAP1 reveal wide variation in expression levels and separate expression. Values are relative fluorescence units. D) The pair-wise correlation between expression values measured for RNA-FISH markers RAP1 and EXP2 reveal wide variation in expression levels and separate expression. Values are relative fluorescence units.

First, we determined the percentage of parasites expressing each marker at each stage. As expected for genes in clusters 1 and 3, SERA4 and RAP1 showed a stage-specific expression (Fig 6A). The percentage of parasites expressing SERA4 began to increase dramatically in middle trophozoites and peaked in late trophozoites, with a majority of late trophozoites expressing this transcript. As the percentage of SERA4-expressing cells decreased markedly in early schizonts (Fig 6A), RAP1 expression peaked and was expressed in nearly all parasites at this stage. Next, we looked at EXP2 expression. Consistent with the previous expression data, EXP2 exhibited a variable and more complex gene expression pattern through the IDC (Fig 6A). It was the only marker expressed in merozoites (Fig 6A and 6B). In addition, EXP2 was also expressed highly in early trophozoites as well as late dividing schizonts (Fig 6A and 6B). Therefore, the expression of cluster-specific marker genes followed a cluster 2, cluster 1, and cluster 3 progression.

Among individual parasites, the expression of SERA4 and RAP1 generally did not overlap, with dominant expression of either SERA4 or RAP1 in each cell (Fig 6B and 6C). This indicates stage-specific expression of SERA4 and RAP1 in each cell with a distinct transition, a pattern also observed in a previous scRNA-seq study [17]. Such a distinct transition between stages differs from the previously understood smooth transition during progression of the IDC [2] as seen in bulk-cell datasets (S5 Fig). For instance, RAP1 is highly expressed starting at 32 hours post-invasion through 4 hours after reinvasion, spanning around 20 hours, as shown in previous microarray data (S5 Fig). However, our RNA-FISH analysis suggests that its expression primarily occurs only in schizonts 38–44 hours post-invasion (Fig 6A and 6B). Additionally, in late schizonts, the expression of EXP2 was separate from RAP1, which was expressed in schizonts more than four hours from bursting (Fig 6A, 6B and 6D).

**Fig 7:**
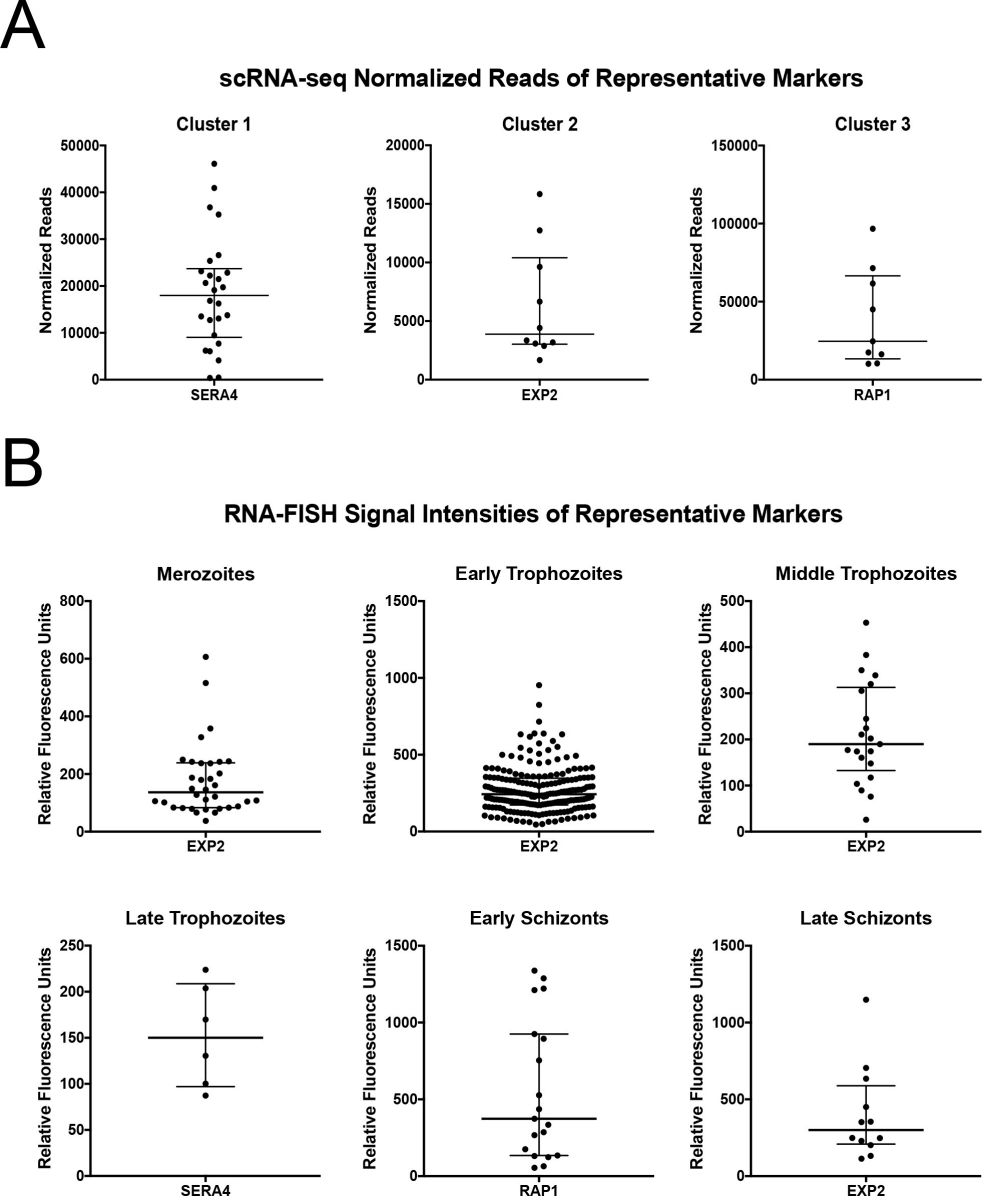
Single-cell analyses indicate wide variation among expressing cells for markers SERA4 (cluster 1), EXP2 (cluster 2), and RAP1 (cluster 3). A) Individual parasite single-cell transcriptomes were assessed for their normalized reads of marker genes representative of each cluster, which showed wide variation in the number of counts per cell. Only expressed values are shown. Error bars represent the median with the interquartile range. B) Individual parasites were measured for their mean fluorescence intensity representative of RNA expression for each marker, which showed wide variation in the expression levels among the exclusive marker-expressing parasites during each stage. Error bars represent the median with the interquartile range.

When visualizing the scRNA-seq data, marker genes from all three clusters exhibited unexpected large variations in expression levels (Fig 7A). For stage-specific markers SERA4 and RAP1, the normalized reads ranged from 377.8 to 46,103.5 for SERA4 in cluster 1 parasites and 10,223.9 to 96,706.5 for RAP1 in cluster 3 parasites (Fig 7A). When we performed a variance analysis across all parasites, we found a similar result, as SERA4 and RAP1 had large biological variations (S3 Table). These wide variations in transcript levels were validated via RNA-FISH for each stage (Figs 7B, S6 and S7). For instance, the relative fluorescence units of SERA4 in late trophozoites with only SERA4 expression (of the three markers tested) ranged from 87.4 to 223.8 (Fig 7B), indicating a wide range of transcription. To normalize this RNA-FISH data, we chose a gene called actin I with low variance across all parasites from scRNA-seq (S3 Table). As we could only test three markers at once with RNA-FISH, we tested SERA4 and RAP1 along with the actin I control. Late trophozoites only expressing SERA4 and actin I showed a distribution of 100.9 to 258.6 relative fluorescence units for SERA4 (S6A Fig) and the pattern and distribution is similar when normalized to actin I (S6C Fig). Similarly, the expression levels of RAP1 assessed by RNA-FISH in individual early schizonts with only RAP1 expression ranged from 54.6 to 1338.0 relative fluorescence units (Fig 7B). Such individual heterogeneity was further validated with the comparison to actin I, showing that the distribution of RAP1 ranged from 117.2 to 2048.2 relative fluorescence units with normalized values keeping a broad distribution (S6B and S6D Fig). Across hundreds of parasites in multiple stages, this stage-specific and heterogeneous pattern holds true (S7A and S7B Fig). Compared to other stages, late trophozoites exhibit greater SERA4 expression and early schizonts exhibit greater RAP1 expression, with variability in their marker expression (S7A and S7B Fig). Such wide variations of stage-specific SERA4 and RAP1 expression among individual parasites may represent an additional level of regulation not seen in previous analyses. However, since the parasite staging by visualization inspection of morphology is intrinsically imprecise, it is also possible that the apparent varying expression levels may result from minor differences in IDC progression of individual parasites.

Our variance analysis also indicated EXP2 as a biologically variable gene, though not to the same extent as RAP1 and SERA4 (S3 Table). Among the sequenced cluster 2 parasites, there was dramatic and large individual variation in the levels of EXP2 expression, which ranged from 1669.8 to 15,843.8 normalized reads in scRNA-seq (Fig 7A). Consistent with this data, EXP2-expressing late schizonts showed a distribution of 113.8 to 1149.1 relative fluorescence units (RNA-FISH) (Fig 7B). Such variability in gene expression among individual cells was undetected at a population level, and could indicate the existence of persistently low or high EXP2 expressers within a population or quick bursts of transcription in small numbers of cells relative to the larger population. This trend is in line with wide variability in SERA4 and RAP1 expression (Figs 6C, 7A, 7B, S6A–S6D, S7A and S7B). Collectively, these data indicate that there are discrete and distinct transitional changes in the transcriptional program during late stage asexual intraerythrocytic development, a phenomenon previously masked in population analyses due to noise from other cells. An understanding of this unusual and hidden transcriptional timing is fundamental to our knowledge of *P*. *falciparum* transcriptional regulation and expression, especially as it relates to malaria drug targets.

## Discussion

Here we applied the Fluidigm C1 microfluidic system and Smart-Seq to perform scRNA-seq of *P*. *falciparum* parasites during their asexual and sexual stages. Through scRNA-seq, we have identified a large number of gametocyte-specific genes as well as three unexpected sub-populations of asexual parasites with distinct gene expression. Furthermore, we have validated our scRNA-seq data using RT-PCR (gametocyte-specific genes) and RNA-FISH (cluster-specific markers EXP2, SERA4, and RAP1) across multiple time points. Our cluster-specific markers were further validated with transcriptomic data from a previous scRNA-seq study [17]. While the transcriptional programs of *P*. *falciparum* during the asexual IDC are highly choreographed with smooth transitions between stages, our scRNA-seq and RNA-FISH data uncover additional layers of heterogeneity not previously observed in population analyses. First, the percentage of SERA4- and RAP1-expressing parasites corresponds to the expected IDC stages and probably contributes to stage-specific expression seen in bulk-cell analyses. However, even among individual parasites scored as the same stage, the expression levels of SERA4 and RAP1 still vary significantly, with high expressers and low expressers. This diversity in expression levels may be due to parasite-to-parasite heterogeneity or subtle differences in cell cycle progression during the same morphological stage. Additionally, the transition between SERA4 to RAP1 expression of individual parasites does not contain as much overlap as predicted by bulk-cell analyses, consistent with another scRNA-seq paper [17]. As rhoptries are secreted during invasion and SERA proteins are involved in parasite egress [40, 42], it appears that their transcriptional pathways might interfere with each other during the late stages of the asexual cycle. Overall, these findings demonstrate the existence of additional regulatory layers and latent heterogeneity in *P*. *falciparum* populations that have been masked in population analyses. The regulatory mechanisms governing this heterogeneity may provide important novel insights into the transcriptional programs and phenotypic fitness of the parasite population during different stages of the IDC.

Most malaria studies focus on analyzing large populations of cells that can only capture the average gene expression of many parasites. These studies are limited in their ability to detect rare populations and heterogeneous gene expression. With the recent availability of various single-cell based methods, scRNA-seq has been applied in different biological contexts to reveal cellular heterogeneity, identify rare cell types, and unravel developmental pathways. Recently, single-cell approaches have been applied to *P*. *falciparum*. We previously used the Fluidigm C1 microfluidic system for single-cell qPCR of annotated male- and female-specific genes, where we uncovered a large number of sex-specific transcripts involved in gametocyte development [27]. Other studies have done scRNA-seq on other various platforms. In one study, analysis of thousands of single-cell transcriptomes positive for AP2-G expression identified gene signatures and additional regulators of sexual commitment [14]. A similar study using LysoPC depletion involved analysis of hundreds of AP2-G positive parasites and also identified a gene signature of sexual commitment, although only three genes overlapped with the previous study [15]. Another paper optimized Smart-Seq2 for use in *P*. *berghei* and *P*. *falciparum* populations, using pseudotime and comparison with bulk-cell datasets to order the single-parasite transcriptomes into distinct clusters [17]. Another described a capillary-based platform for single-cell isolation and uncovered novel genes upregulated in early stage gametocytes with a modified Smart-Seq2 protocol [16]. The latest paper used 10x Genomics’ droplet system on thousands of parasites, including field samples, to generate single *Plasmodium* transcriptomes across the complete life cycle [18].

Our scRNA-seq analysis generally agrees with previous studies but also reveals some novel insights. First, our comparison of asexual and sexual parasites identified multiple gametocyte-specific genes that were not described in previous proteomic, microarray, or sequencing studies [5, 25, 26, 43], including eight that were validated by qPCR. Of these eight, four have unknown functions and the other four putatively include a peptidase, kinesin, RAP protein, and AAA family ATPase, the most highly induced. Further investigation is needed to elucidate their roles in gametocytogenesis and sexual reproduction. Second, our data suggests a more complicated expression pattern during schizogony, with distinct transitions from SERA to rhoptry expression and then rhoptry to EXP2 expression, previously masked in population analyses. Other scRNA-seq studies also show these distinct transitions by using pseudotime analysis [14, 17]. In one study, expression of microneme and inner membrane complex (IMC) genes followed rhoptry expression, but this was only captured in *P*. *berghei* and not *P*. *falciparum* [17]. Drop-seq of thousands of *P*. *falciparum* parasites was able to capture the subsequent expression of microneme and IMC genes as well as CDPK5 after rhoptry expression in *P*. *falciparum* [14]. In contrast to these studies, our RNA-FISH data, together with morphological staging, have independently verified and ordered cells according to their gene expression. In particular, this approach showed that EXP2 is expressed at multiple times throughout the life cycle, and similar validation methods may be used to analyze transcripts with non-conventional expression patterns uncovered by scRNA-seq analysis.

One unexpected finding in our study is the existence of a wide distribution in the number of transcripts expressed among different marker-expressing parasites of similar IDC stages determined by visual inspection. Such cell-to-cell variations in the expression level are most strikingly seen in schizonts expressing either RAP1 or EXP2 in our RNA-FISH dataset. Cell-to-cell expression variability in these genes/proteins may result from intrinsic differences between individual parasites or slight differences in cell cycle and differentiation progression. It is important to note that parasite staging by visualisation by light microscopy is inherently imprecise. Therefore, one possible explanation is that scRNA-seq and RNA-FISH may reveal the latent heterogeneity not observable using visual staging methods. Regardless of the underlying mechanisms, such variations may be relevant for the interpretation of many malaria experiments based on stages. These variations may lead to significant functional heterogeneity among different parasites and enhance the overall population fitness, a possibility that may be studied with fluorescent reporter strains of parasites and single-cell imaging. For instance, this heterogeneity may contribute to *P*. *falciparum*’s ability to adapt to various stresses, different host environments, and drug treatments, with some parasites better equipped to handle fluctuations and/or stresses than others. Recently, it was shown that higher expression levels of EXP2 are correlated with higher numbers of merozoites produced per dividing schizont, indicating that EXP2 is important for parasite fitness [32]. Perhaps such high expressing cells exhibit other phenotypes such as an enhanced invasion ability or a proclivity towards drug resistance. Likewise, the varying expression of MDR1 may allow a sub-population of parasites to survive drug treatment. Such clonal fitness under stress may help to explain the association of MDR1 expression with the emergence of drug resistance. Similarly, rhoptry proteins of *Plasmodium* are implicated in the invasion of new host RBCs. The cell-to-cell variations in the levels of rhoptry expression may affect the parasite’s invasion ability and the selection of RBCs to invade, including new versus old cells. Therefore, such cell-to-cell variations of gene expression may enhance the adaptability of the parasite population to maintain homeostasis as it goes through different host environments and a wide variety of stresses. Thus, these huge cell-to-cell variations in the expression levels could have implications for drug development and resistance, immune pressures, and malaria disease severity and clinical outcomes.

Importantly, as sexual reproduction only occurs in the mosquito, these asexually replicating haploid cells putatively form clonal populations with little genetic diversity. Furthermore, all of the analyzed single parasites come from a population that has been cultured in the same media with identical conditions. Even with these identical genetic and environmental factors, our data clearly indicate that there are incredible levels of cell-to-cell variation which have not been recognized in previous population analyses. Therefore, elucidating the genetic basis and sources of cell-to-cell variations will be of high interest.

First, it is possible that there are epigenetically controlled mechanisms that enhance or repress stage-specific gene expression programs in each individual parasite. Such epigenetic regulation of expression levels in *P*. *falciparum* has been described for *var* genes [44, 45] and sexual commitment [9]. Additionally, genetically homogenous parasite populations can be epigenetically highly heterogeneous, with transcriptionally diverse populations having the ability to quickly adapt to stress [46]. A previous study has also shown that transcript level variation is strongly influenced by parasite genotype and can be controlled by several regulatory hotspots in the genome, including a region associated with multiple drug resistance [47]. Transcriptional heterogeneity also results from sporadic bursts of gene expression in a small number of parasites that is transient and not passed along to the next generation. Finally, it is possible that cell-to-cell expression variations may also result from the intrinsic and extrinsic genetic noise noted in other systems [48]. RAP1 and other highly variably expressed genes can be used to investigate the underlying mechanisms and potential contributions of transcriptional bursts or genetic noise.

We envision that scRNA-seq combined with RNA-FISH will have broader applications in the parasitology field. While scRNA-seq can provide insight into the many genes expressed in a single parasite, including rare expression, RNA-FISH can be used to identify the spatial distribution of specific transcripts and quantify their levels in individual cells. When combined with morphological analysis, they can be used to uncover distinct but rare parasite phenotypes that may be major contributors of disease. We anticipate that such single-cell approaches can be used to determine gene expression in malaria-infected RBCs in patients during the natural progression of the disease or during drug treatment. We can identify the rare drug-resistant parasites that emerge during drug treatment due to their fitness advantage and devise strategies to prevent drug resistance. Given the small number of parasites committed to sexual differentiation, another obvious application of scRNA-seq is to define the differentiation hierarchy and underlying molecular mechanisms of sexual differentiation into male and female gametocytes to identify mating-type determination [14, 15, 27]. In addition, since RBCs also have RNA transcripts [49–51] with the potential for regulating malaria infection [52, 53] and intercellular communication [54], it will be interesting to perform parallel transcriptome profiling of the host RBCs and infecting parasites to identify novel genetic determinants of invasion. Similar approaches can also be used to dissect host-pathogen interactions in hepatocyte and mosquito host cells.

## Materials and methods

### Parasite strain and culture

*P*. *falciparum* strain 3d7a was obtained from the Malaria Research and Reference Reagent Resource Center (MR4). Using standard procedures, parasites were cultured in 30 mL of RPMI 1640 medium supplemented with 0.5% AlbuMAX II at 2% hematocrit in human B+ erythrocytes at 3% O_2_/5% CO_2_ [55, 56].

### Culture synchronization and gametocyte induction

To synchronize cultures, parasites were treated with 5% D-sorbitol twice, approximately 48 hours apart during the ring stage [57]. The synchronization was verified by Giemsa stained blood smears. To enrich for gametocytes, 50 mM N-acetyl glucosamine was added to the culture on the day following the second 5% D-sorbitol treatment. This addition of N-acetyl glucosamine to cultures with a parasitemia between 6–10% was considered day 0, with drug treatment continuing for a total of 72 hours with daily medium changes [58]. Gametocyte production was monitored with Giemsa stained blood smears. On day 9, before gametocyte collection, the gametocyte culture was treated with 5% D-sorbitol to eliminate any contaminating trophozoites.

### Purification of individual parasites

A 40/70% Percoll density gradient was used to either isolate late stage asexual (approximately 34 hours post-invasion) or day 9 gametocytes from rings, early gametocytes, and uninfected erythrocytes [58]. To eliminate all uninfected erythrocytes, the sample collected from the 40/70% interface was run through a MACS LS column, yielding pure trophozoite/schizont and gametocyte populations. The isolated cells were pelleted by centrifugation and resuspended to a concentration of at least 2.5x10^5^ cells/mL in complete malaria media.

### Single-cell capture and sequencing

To determine the viability and concentration of the isolated cells, 10 μL of cells were mixed with 10 μL of trypan blue stain (0.4%) and loaded onto a Countess Automated Cell Counter (Thermo Fisher Scientific). Viable cells were adjusted to concentrations of 3.2x10^5^ cells/mL for gametocytes and 4.4–4.9x10^5^ cells/mL for late stage asexual parasites. According to Fluidigm protocol, 60 μL of gametocytes were mixed with 40 μL of Suspension Reagent (Fluidigm) before being loaded onto a C1 Single-Cell Auto Prep Integrated Fluidic Circuit (IFC) for mRNA-seq (5–10 μm). We noticed that asexual parasite capture rates at this ratio were low, and upon further inspection, saw that cells at this concentration fell to the bottom on the cell inlet, indicating that the asexual parasite concentration was too dense. To optimize the cell buoyancy and improve capture, we tested various ratios of cell number to suspension buffer using a concentration of 400 cells/μL. We found that 40 to 50 percent of cells making up the final suspension buffer was optimal, although capture rates were still low at 24 to 27 percent (compared to late-stage gametocyte capture rates of 77 percent in this study and 92 percent in a previous study [27]). Therefore, we used two C1 Single-Cell Auto Prep IFCs to capture 46 synchronized asexual parasites on two different days.

For scRNA-Seq library construction, reagent mixes were prepared according to the Fluidigm protocol (PN 100–7168 L1). The IFC was primed and loaded with 6 μL of cell mix according to manufacturer’s instructions, using the mRNA Seq: Prime (1771x) and mRNA Seq: Cell Load (1771x) scripts. Once the cells were loaded, each well of the IFC was checked for the presence of a single parasite under a microscope. Harvest reagent, lysis mix, RT mix, and PCR mix were added to the IFC according to Fluidigm protocol, with 1 μL of ERCC spike-in sequences (Thermo Fisher Scientific, 4456740) added to the lysis mix at a 2.5 x 10^5^ dilution of the original product. The IFC was then loaded into the C1 system, running the mRNA Seq: RT & Amp (1771x) script overnight. Amplified products were harvested and diluted, with approximately 3 μL of amplicons diluted in 10 μL of C1 DNA Dilution Reagent (Fluidigm).

Population controls for two asexual and one gametocyte population were prepared in parallel with the C1 single-cell capture according to “Appendix A: Run the Tube Controls”. “Protocol A: Tube Controls with Purified RNA” was followed. Samples were vortexed for one minute for the homogenization step, and a gDNA Eliminator spin column was used for the removal of contaminating genomic DNA. When diluting the final products after amplification, 40 μL of C1 DNA Dilution Reagent (Fluidigm) was used to every 1 μL of PCR product.

The Nextera XT kit was then used to generate the sequencing library from the cDNA according to the Fluidigm protocol. Once the libraries were quantified, they were sequenced in one lane on an Illumina HiSeq 2500 with 50 bp single-end reads. 10% PhiX was used as a spike-in control for *P*. *falciparum*’s highly AT-rich genome. All sequencing data is available at NCBI, SRA accession number SRP151825.

### Generating gene counts

Single-cell RNA-seq data was first processed using the TrimGalore toolkit [59] which employs Cutadapt [60] to trim low-quality bases and Illumina sequencing adapters from the 3’ end of the reads. Only reads that were 20nt or longer after trimming were kept for further analysis. Using the STAR RNA-seq alignment tool [61], reads were mapped to the *P*. *falciparum* 3D7 genome and transcriptome sequence (PlasmoDB r13.0), as well as the GRCh37v75 version of the human genome and transcriptome to filter out human RNA originating from the blood sample. Reads were kept for subsequent analysis if they mapped to a single genomic location of the *P*. *falciparum* genome. Gene counts were compiled using the HTSeq tool [62].

### Cell and gene filtering

QC filtering was performed using the R package scater v1.4.0 [63]. As expected, the number of expressed genes for bulk RNA controls was higher (between 4,000 and 5,000) than for sequenced single cells (between 1,200 and 3,600) (Fig 2). Principal component analysis (PCA) based on phenotypic data such as library size, number of expressed genes or spike-in (ERCCs) proportion, did not expose any outliers out of the 51 cells that were sequenced. To filter out low-abundance genes, only genes with average counts at least equal to 1 were kept. 4,694 genes remained after gene filtering when considering all 51 cells, and 4,463 remained when considering the 46 non-gametocyte cells.

### Normalization of gene expression

To normalize gene expression, size factors were calculated using the R package scran v1.4.5 [64]. The package scran estimates cell-specific biases by deconvolving size factors using cell pools. The assumption is that most genes are not differentially expressed between cells. Expression values were normalized using the previously generated size factors then log-transformed. Normalization was performed independently on two sets of cells: the whole 51 cells, and the 46 non-gametocyte cells. All downstream applications were performed using normalized gene expression. The normalized gene expression is available in S1 Table (51 cells) and S2 Table (46 cells).

### Clustering

To perform clustering, we used the R package SC3 v1.4.2 [24]. After selecting genes that are expressed in at least 10% of cells but also no more than 90% of cells, *SC3* performs k-means clustering on distance matrices between cells that have been transformed with either principal component analysis (PCA) or Laplacian eigenvectors. *SC3* combines the obtained clustering solutions into a consensus matrix that summarizes how often two cells are part of the same cluster. The final clustering solution is given by the complete-linkage hierarchical clustering of the consensus matrix into k groups. Prior to clustering, *SC3* can estimate an optimal number of clusters using the Tracy-Widom theory on random matrices. When clustering the 51 cells, SC3 estimated the optimal number of clusters to be four, with the five gametocytes forming a stable cluster of their own as measured by its silhouette width of 1. A silhouette width is a quantitative measure between 0 and 1 that describes the cohesion within a cluster, as well as its separation from other clusters, with the best clustering occurring when the average silhouette width is close to 1. The silhouette widths of the three other clusters were 0.51 (cluster 1), 0.81 (cluster 2) and 0.69 (cluster 3) (S8A Fig). To analyze the asexual cells independently, we also performed clustering of the 46 non-gametocyte cells. Consistent with the first clustering analysis, SC3 suggested three clusters, almost identical to the asexual clusters defined in the four-cluster analysis (only three cells clustered differently). Their silhouette widths were 0.49 (cluster 1), 0.88 (cluster 2) and 0.71 (cluster 3) (S8B Fig). Both datasets with gametocytes and without gametocytes were subjected to PCA analysis for visualization of the clusters (Figs 3A and 4A).

### Identification of markers

To identify candidate marker genes for each cluster, we used SC3 to evaluate the potential for each gene to be a marker for the cluster in which its mean expression value was the highest. For each cluster, we retained genes with a non-zero probability of being a marker based on a Wilcoxon signed rank test. We then ordered these candidate markers by decreasing area under the receiver operating characteristic (AUROC) for further validation. This was done both for the five gametocytes versus all other cells in the four-cluster analysis, and for each cluster in the three-cluster analysis.

### Variance analysis of gene expression

To analyze variance for each gene in the scRNA-seq dataset, the R package *scran* was used to fit a mean-variance trend to the spike-in transcripts (S3 Table). The trend helps quantify the technical component of the variance. The biological component for each gene is then deduced as the difference between the total variance and the technical component.

### Inferring a cell trajectory

To generate a cell progression hypothesis, we used the R package *monocle* v2.8.0 [24]. A differential expression test was performed for each gene using the clusters as indicator variables (full model ~cluster and reduced model ~1) to detect genes expressed differentially between clusters. As a result, 84 genes with a q-value from the likelihood ratio tests < 0.01 were chosen as genes to order and infer a trajectory. We then used the DDRTree dimensionality reduction algorithm from *monocle* to align the cells on a branched path, as shown in Fig 4C. The trajectory includes a main path and two very small branches. To confirm this analysis, we also ran *monocle* without using clusters as an indicator variable for selecting genes to represent cell progress. Two additional analyses were run, each using a different set of genes: 1) highly expressed genes with an average gene expression ≥ 10, and 2) genes with high dispersion across cells based on the expected mean-dispersion calculated by *estimateDispersions*. Both trajectories were consistent with the clustering and ordering of cells calculated using *SC3* clusters as indicator variables. However, both additional analyses supported a larger independent branch for a portion of cells from cluster 1 (S2 Fig). Because cluster 2 markers are expressed the earliest [2], cluster 2 cells were used to define a start for all cell trajectories and pseudotime calculated by *monocle*.

To identify genes that co-vary across pseudotime, we used the lineage analysis based on highly expressed genes and focused on the main trajectory. The analysis was independent from our previous *SC3* clustering. Using the *branchTest* function in *monocle*, we identified genes that are differentially expressed between branches originating from both branching points (black circles 1 and 2 in S2 Fig). We restricted the two resulting gene lists to genes with a q-value < 0.01 and obtained 141 unique genes from the union of these two lists. We used the *plot_pseudotime_heatmap* function in *monocle* to visualize genes which expression varies around branch points 1 and 2, and restricted cells to the 32 cells on the main trajectory (S3 Fig). The heatmap displays smooth expression curves and both gene expression and pseudotime are binned to 100 columns.

### Assessing bias towards longer transcripts

Transcript length was deduced from the *P*. *falciparum* 3D7 PlasmoDB r13.0 transcript sequences. To assess the effect of transcript length, we compared the length distribution of transcripts in the initial dataset (n = 5,452) to that of “expressed genes” remaining after filtering of genes with an average gene count below 1 (n = 4,694).

### Validation of asexual clustering

To validate the clustering of the asexual cells into three clusters, we clustered the *P*. *falciparum* asexual scRNA-seq dataset available from Reid et al. [17] using the 151 markers identified from the clustering of our asexual dataset. We processed the raw count tables the same way as ours. Using the R package *scran*, we detected eight outlier cells that we removed from the dataset, and filtered genes with an average count below 1. We then normalized the resulting dataset that consisted of 4,844 expressed genes and 172 samples. All 151 asexual markers identified earlier were expressed in the Reid et al. asexual dataset and the gene space of the dataset was reduced to these 151 genes for clustering. *SC3* estimated the optimal number of clusters to be three with an average silhouette width of 0.72. After testing the 151 genes as markers for these three clusters with similar thresholds, we compared our three clusters to the obtained three Reid et al. clusters based on shared markers (S6 Table).

### Bulk-cell qPCR

RNA was extracted from synchronized late stage asexual parasites and day 9 gametocytes using TRIzol Reagent (Invitrogen). The RNeasy Mini Kit (Qiagen) was used to remove DNA by DNase treatment according to Appendix E: DNase Digestion of RNA before RNA Cleanup. cDNA was synthesized from 50 ng of RNA using SuperScript II Reverse Transcriptase (Invitrogen) and 50 ng of random hexamers (Bioline). Primers (Eurofins Genomics) were designed for 8 novel gametocyte-specific genes (S4 Table), which were tested for their gene expression differences using Power SYBR Green (Invitrogen) real-time PCR. Pfs16 was used as a control for gametocyte numbers between asexual and day 9 gametocyte samples. The ΔCt value was calculated relative to the constitutive control, tRNA ligase. The ΔΔCt value was calculated relative to the asexual sample, with the expression fold change of 2^-ΔΔCt representing the increase in expression of genes in the gametocyte sample.

### RNA fluorescent *in situ* hybridization

400 μL of a synchronized culture was lysed with 0.15% saponin to isolate single parasites. Briefly, parasites were pelleted and then washed with PBS twice before being resuspended in at least 1 mL of 0.15% saponin. The parasites were then vortexed for one minute before being placed on ice for 20 minutes. The cells were centrifuged at 2100xg for 12 minutes at 4°C and washed once in PBS. After centrifugation, these cells were fixed in 1 mL of 4% formaldehyde for 30 minutes. The tube was vortexed halfway between the incubation to resuspend the cells. “Appendix A: Sample Preparation Procedure for Suspension Cells” of the QuantiGene ViewRNA ISH Cell Assay Kit (Thermo Fisher Scientific) was then followed after the fixation step. 30 μL of poly-D-lysine (Sigma) was used to coat microscope slides, and two hydrophobic barriers were drawn on each slide, one on which probes were added and one in which they were not. Cells were baked on the slides at 50°C for 20 minutes. The assay procedure then continued with the rehydration step, adding the solutions directly to the slide. The probes used were *Plasmodium falciparum* Sera4 (VF4-6000578), Rap1 (VF6-21027), EXP2 (VF1-6000683), and Actin I (VF1-21025), all from Thermo Fisher Scientific. Mounted samples were cured overnight with protection from light and were viewed using a Zeiss Axio Observer 7.1 microscope with a 63x oil objective. DAPI, Cy5, mRFP1.2, and EGFP filters were used to detect DAPI, Cy5, Cy3, and FITC, respectively. The images were processed using Fiji software and individual cells were outlined using the freeform tool and measured for their mean fluorescence signal. The background signal from each image and channel was eliminated from individual cell fluorescence signals. Individual cells were also scored for their stage-specific progression and gene expression, according to previous morphological analysis [41]. Free merozoites were scored after egress from schizonts and were defined by DAPI staining.

## Supporting information

S1 Fig The distribution of transcript lengths from expressed genes (after filtering out genes with an average count below 1) shows slight length bias for longer transcripts when compared to the distribution of transcript lengths from all genes. (PDF)

S2 Fig Pseudotemporal ordering of 46 single asexual parasites by *monocle* 2. Highly expressed genes with an average gene expression ≥ 10 were used for ordering cells and inferring a trajectory. Top panel: cells are colored by pseudotime. Bottom panel: cells are colored by cluster assignment.
(PDF)

S3 Fig Heatmap showing the smoothed, scaled and centered gene expression for 141 genes that show scRNA-seq dynamic expression around branch points 1 and 2 (S2 Fig). Only gene expression in the 32 cells of the main trajectory was used for the heatmap. Cell expression is smoothed and spread across 100 bins distributed uniformly across pseudotime. Genes are grouped by expression pattern.
(PDF)

S4 Fig Consensus clustering matrix of 172 single asexual *P. falciparum* parasites from Reid et al. represents the optimal three clusters estimated by SC3. Parasites within the same cluster show the strongest similarity when compared to parasites from other clusters, although there is significant heterogeneity even between parasites in the same cluster. The color scale indicates the likelihood that two cells are arranged in the same cluster (blue: low (0), red: high (1)).
(PDF)

S5 Fig Hierarchical clustering of the top marker genes for each cluster from the microarray 48-hour time course published in Bozdech et al., 2003. The log_2_ (Cy5/Cy3) ratios for markers of clusters 1 (A), 2 (B), and 3 (C) were centered by the mean and clustered using complete linkage to examine their expression profile during the IDC. The black arrow represents the expected stage of the harvested parasite population for scRNA-seq.
(PDF)

S6 Fig RNA-FISH analyses indicate wide variation among expressing cells for A) SERA4 (late trophozoites) and B) RAP1 (early schizonts). Individual parasites were measured for their mean fluorescence intensity representative of RNA expression for each marker, which showed wide variation in the expression levels among the exclusive marker-expressing parasites. These values were normalized to actin I for C) SERA4 and D) RAP1. Error bars represent the median with the interquartile range.
(PDF)

S7 Fig RNA-FISH analyses reveal variation of A) SERA4 and B) RAP1 when normalized to actin I control. SERA4 shows highest expression in late trophozoites, while RAP1 shows highest expression in early schizonts. Error bars represent the median with the interquartile range.
(PDF)

S8 Fig Silhouette widths of single parasites reflect both how well matched individual cells are to their own cluster (cohesion) and how poorly matched they are to neighboring clusters (separation) for A) 51 sexual and asexual cells, B) 46 asexual cells, and C) 172 asexual cells from a previous dataset [17]. A silhouette width value ranges from -1 to +1, with a high value indicating that the cell is similar to its own cluster but not to its neighboring clusters.
(PDF)

S1 Table Normalized gene expression for 51 cells. (XLSX)

S2 Table Normalized gene expression for 46 cells. (XLSX)

S3 Table Variance analysis across 51 cells. (XLSX)

S4 Table Primers used for bulk-cell qPCR validation of novel gametocyte-specific genes. (XLSX)

S5 Table List of 340 gametocyte-specific genes revealed by scRNA-seq. Annotations for significantly differentially expressed male and female genes were obtained from Lasonder et al. [28].
(XLSX)

S6 Table List of 151 cluster markers shared between our dataset and Reid et al. (XLSX)
