# Supplementary figures and images for: Latent transcriptional variations of individual Plasmodium falciparum uncovered by single-cell RNA-seq and fluorescence imaging
Source: PLoS Genet. 2019 Dec 19;15(12):e1008506. doi: 10.1371/journal.pgen.1008506 (PMC6952112; doi:10.1371/journal.pgen.1008506)

Transcript length distribution

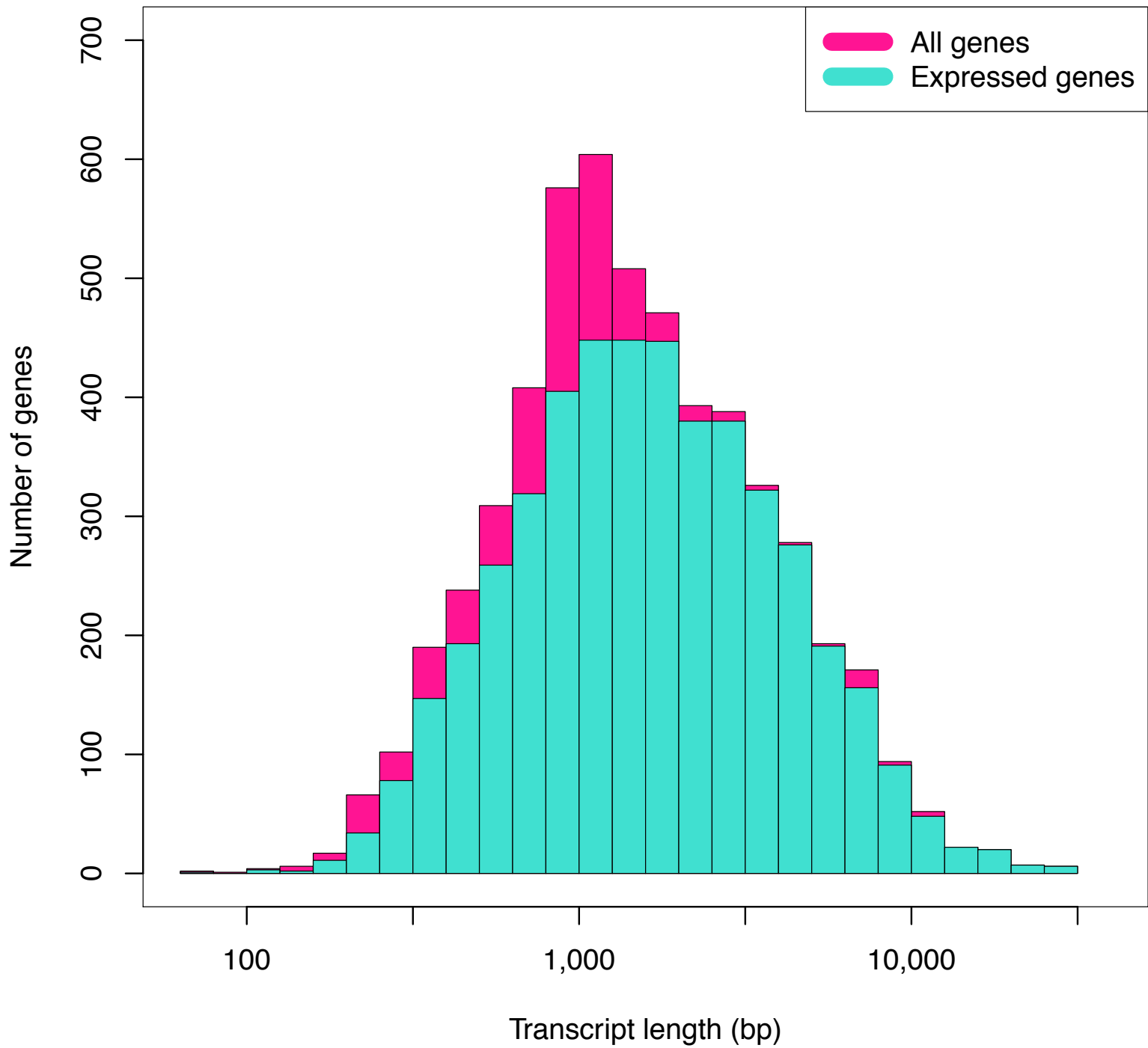

Supplement: S1 Fig — (PDF) [file pgen.1008506.s001.pdf]

# Pseudotime Trajectory Without Clusters

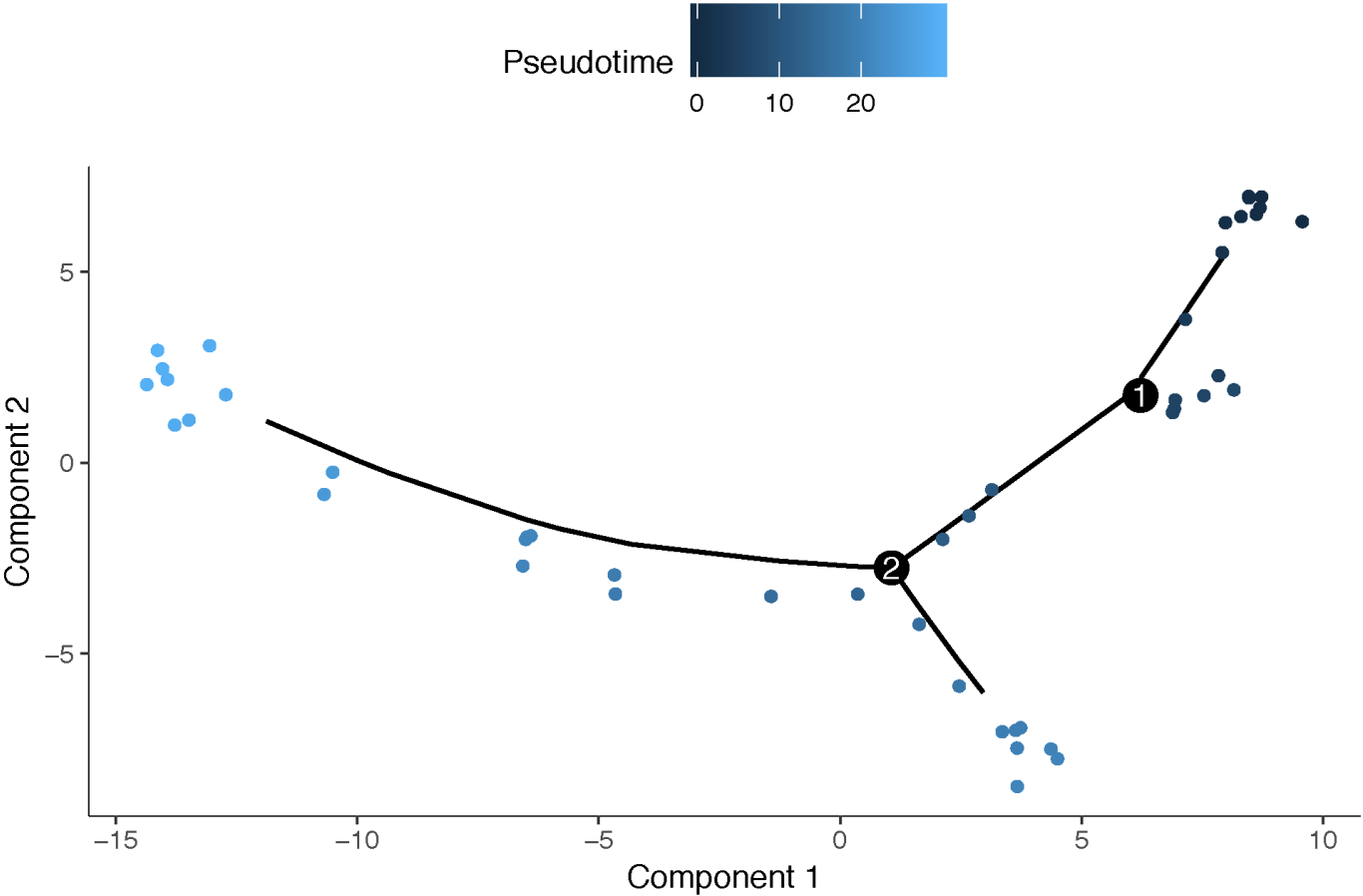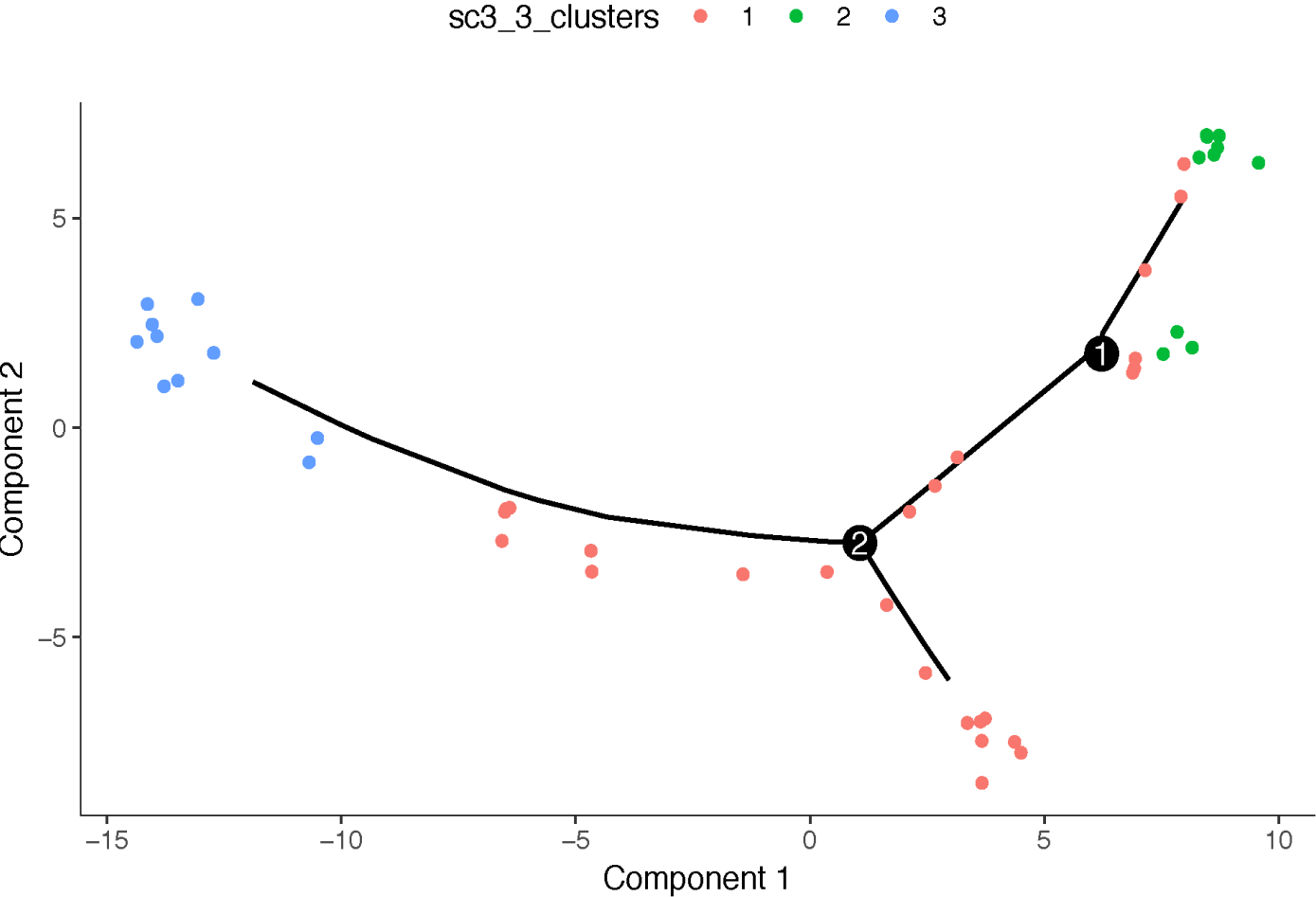

Supplement: S2 Fig — Highly expressed genes with an average gene expression ≥ 10 were used for ordering cells and inferring a trajectory. Top panel: cells are colored by pseudotime. Bottom panel: cells are colored by cluster assignment. (PDF) [file pgen.1008506.s002.pdf]

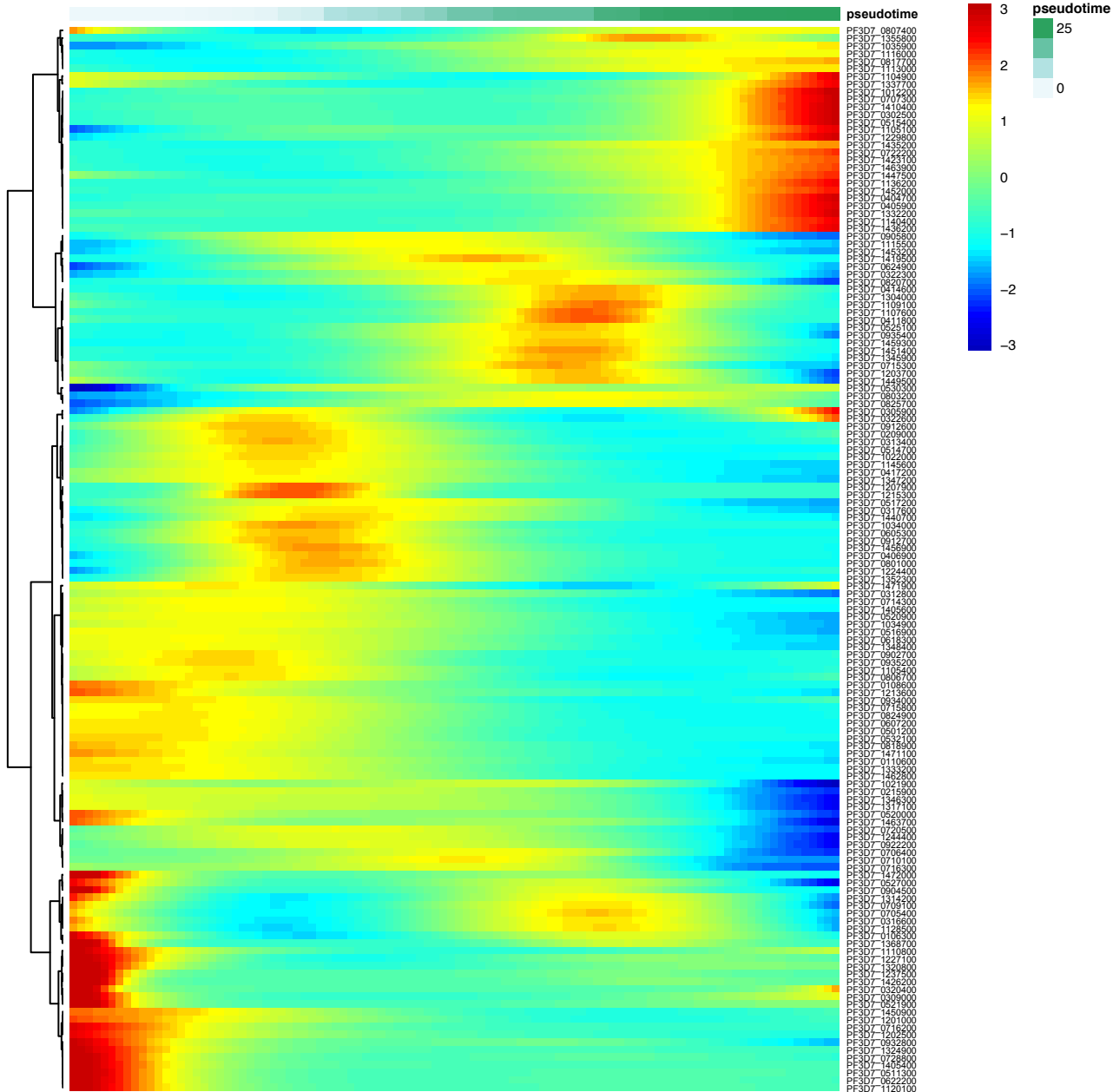

Supplement: S3 Fig — Only gene expression in the 32 cells of the main trajectory was used for the heatmap. Cell expression is smoothed and spread across 100 bins distributed uniformly across pseudotime. Genes are grouped by expression pattern. (PDF) [file pgen.1008506.s003.pdf]

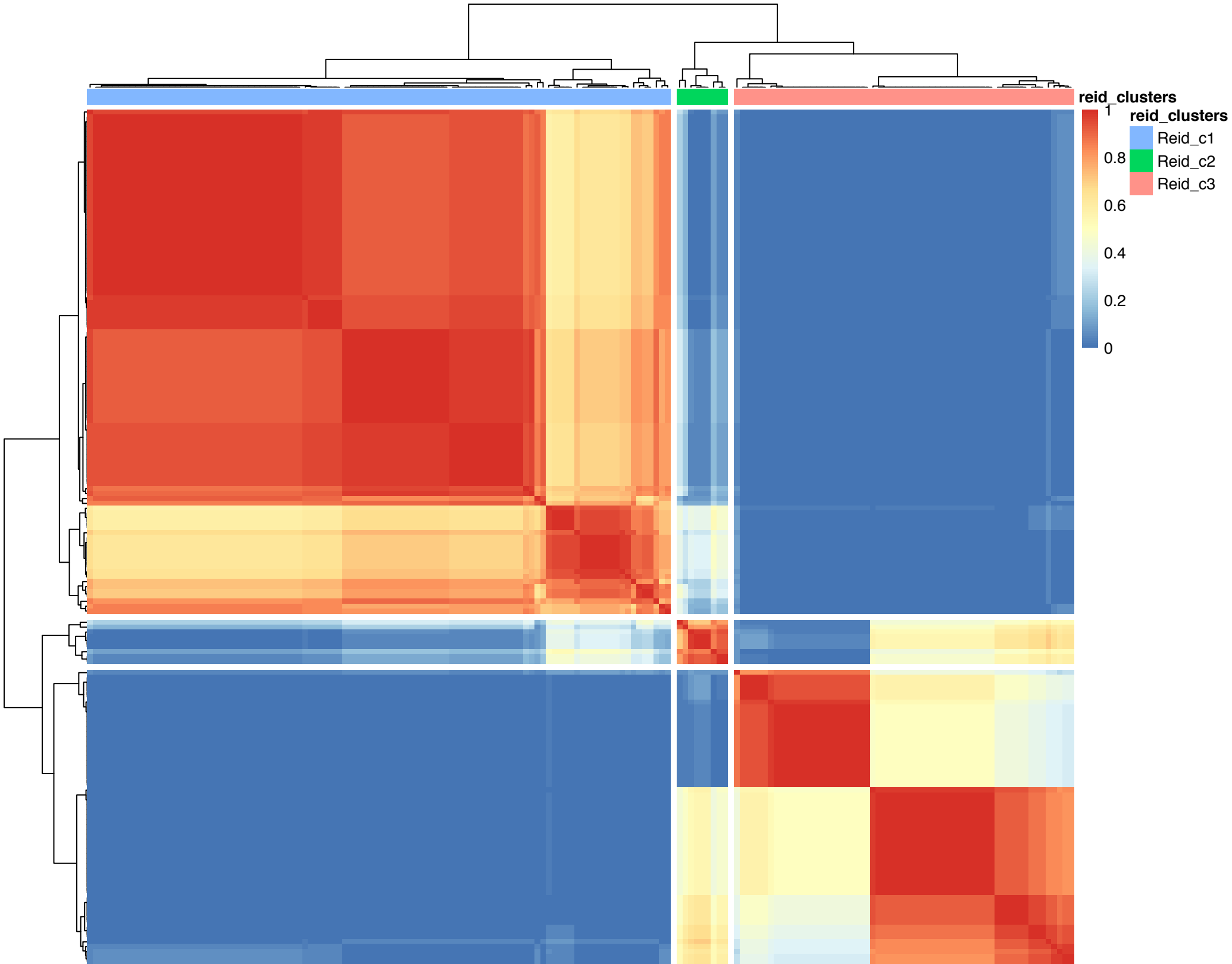

Supplement: S4 Fig — Parasites within the same cluster show the strongest similarity when compared to parasites from other clusters, although there is significant heterogeneity even between parasites in the same cluster. The color scale indicates the likelihood that two cells are arranged in the same cluster (blue: low (0), red: high (1)). (PDF) [file pgen.1008506.s004.pdf]

A

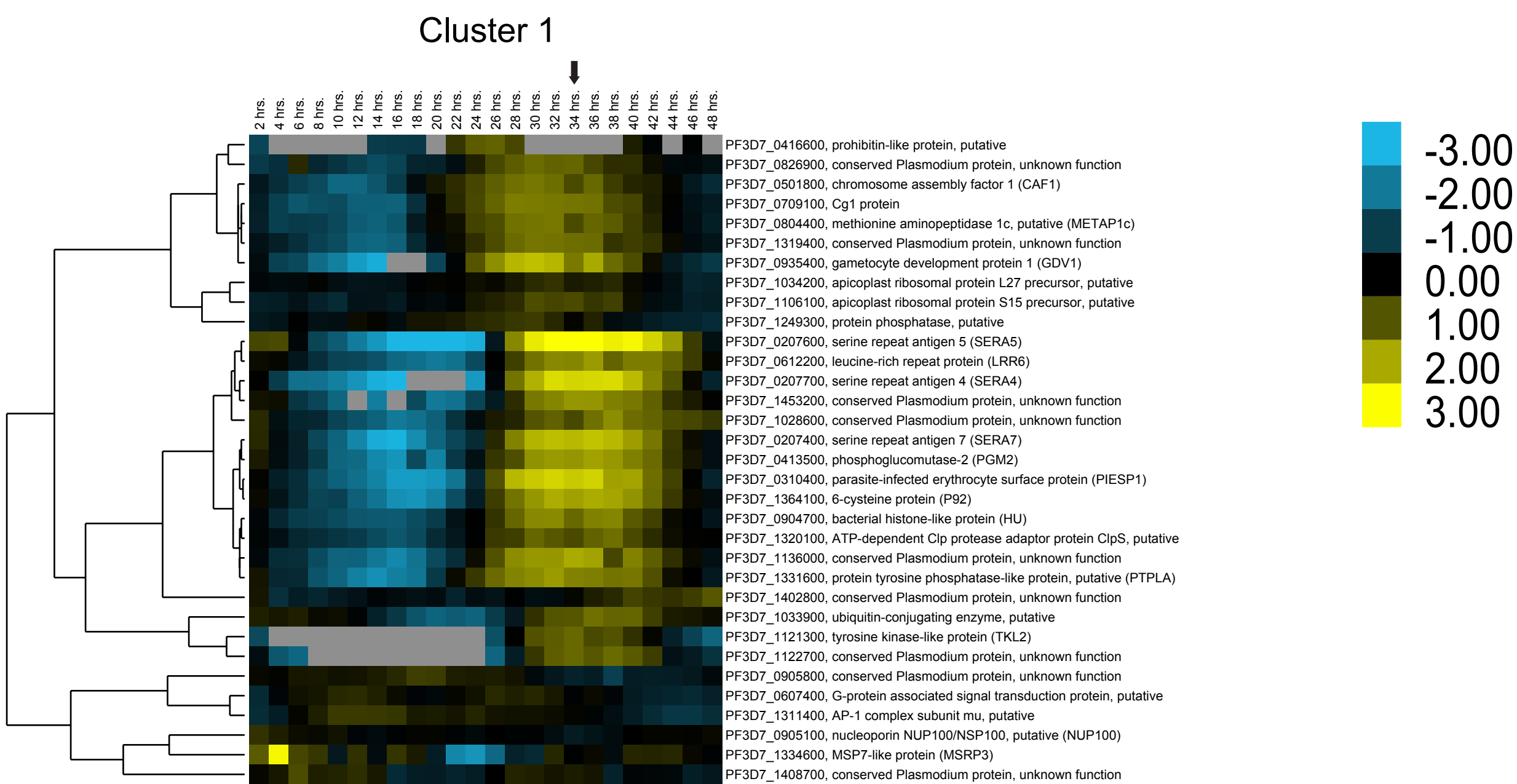

B

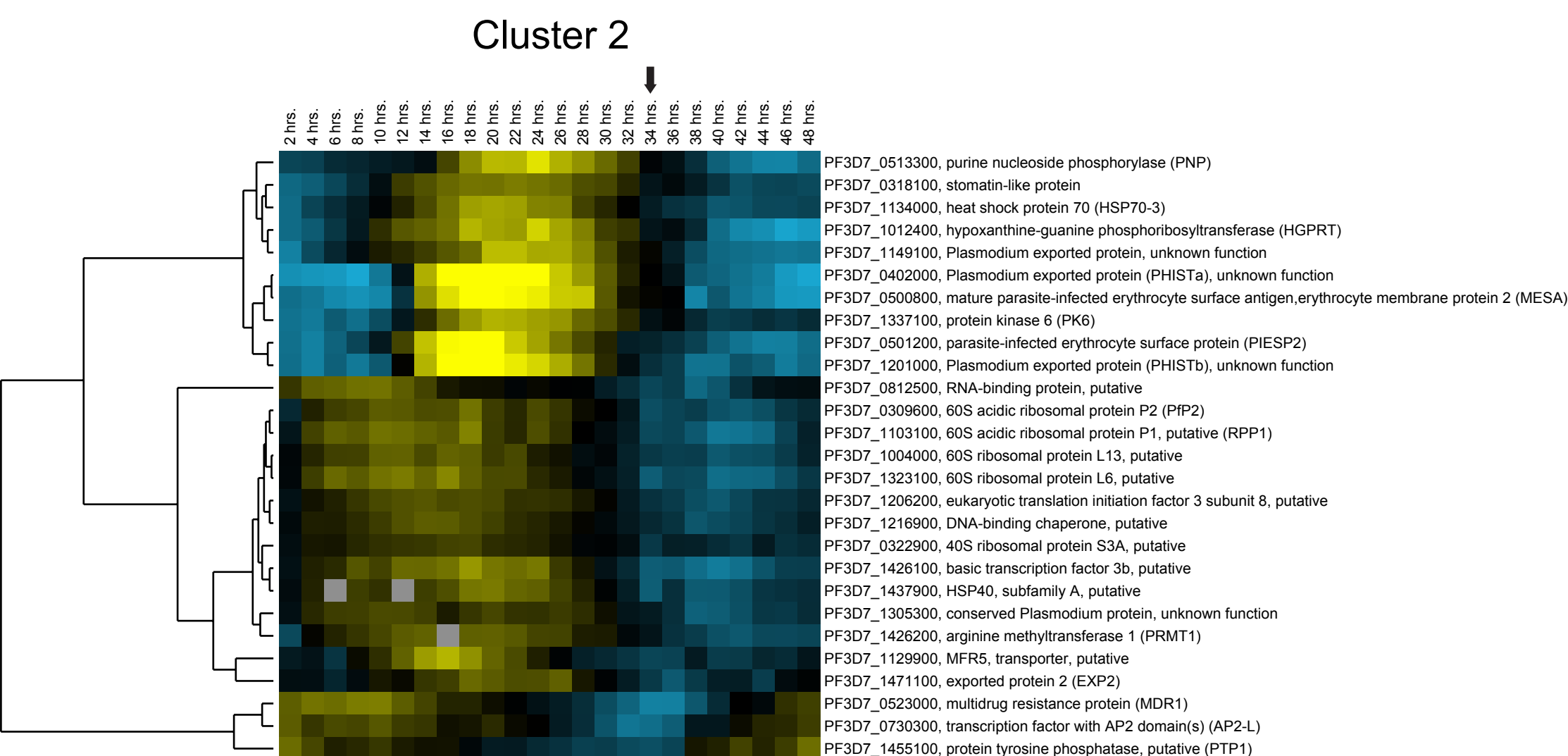

C

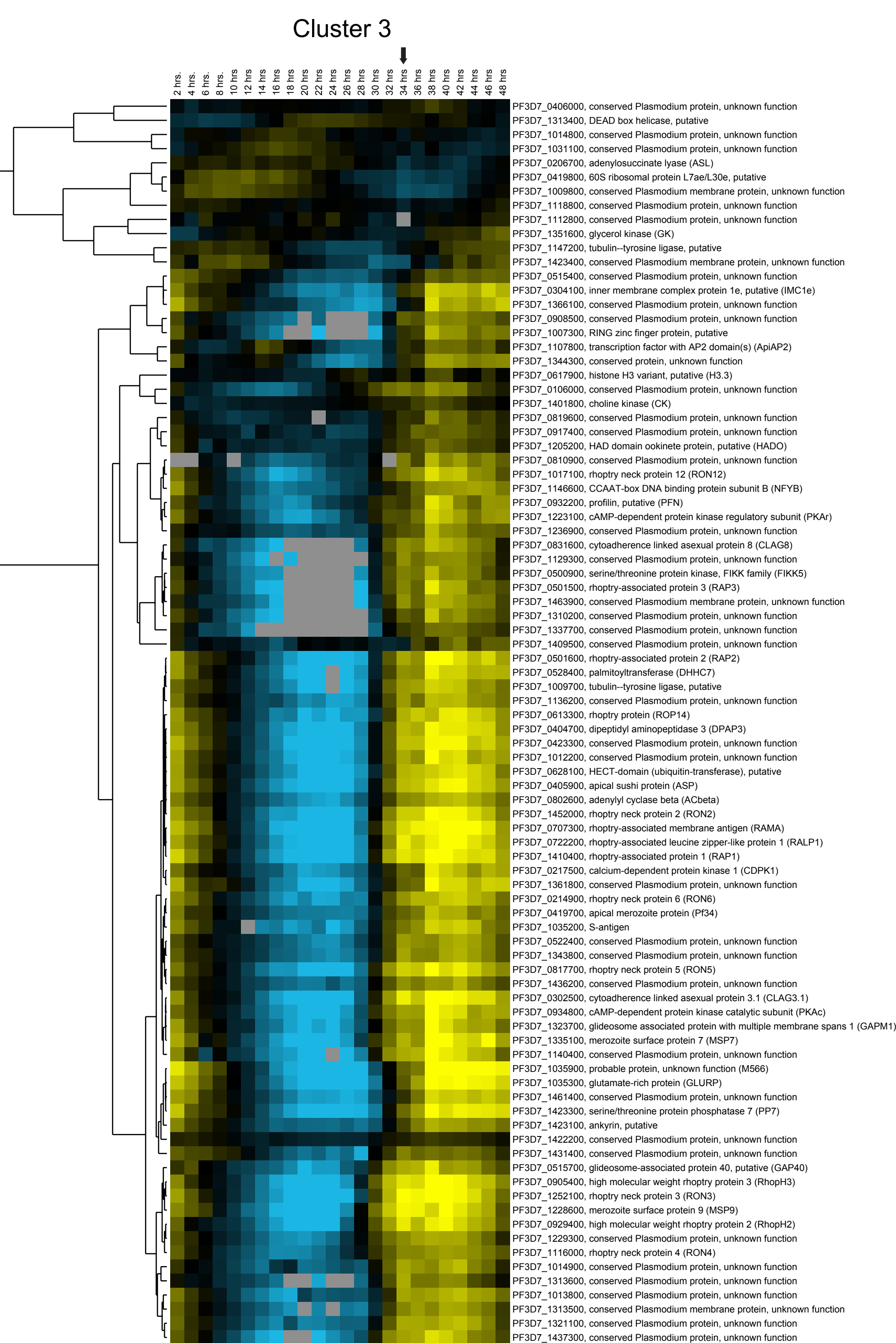

Supplement: S5 Fig — The log2 (Cy5/Cy3) ratios for markers of clusters 1 (A), 2 (B), and 3 (C) were centered by the mean and clustered using complete linkage to examine their expression profile during the IDC. The black arrow represents the expected stage of the harvested parasite population for scRNA-seq. (PDF) [file pgen.1008506.s005.pdf]

**A**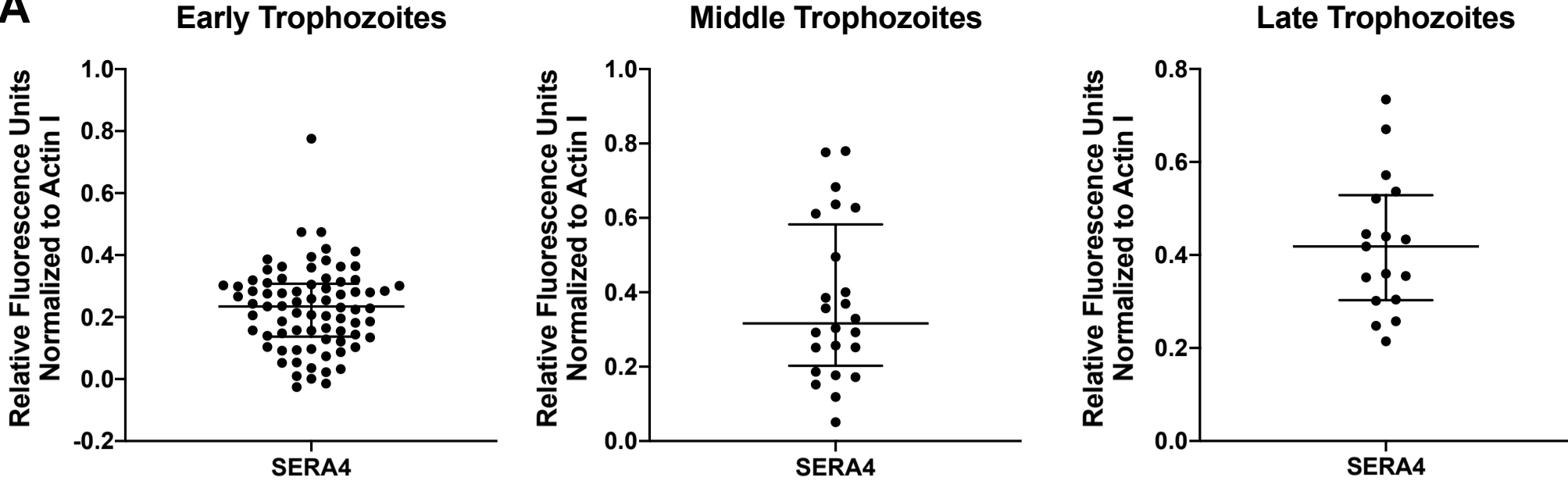**B**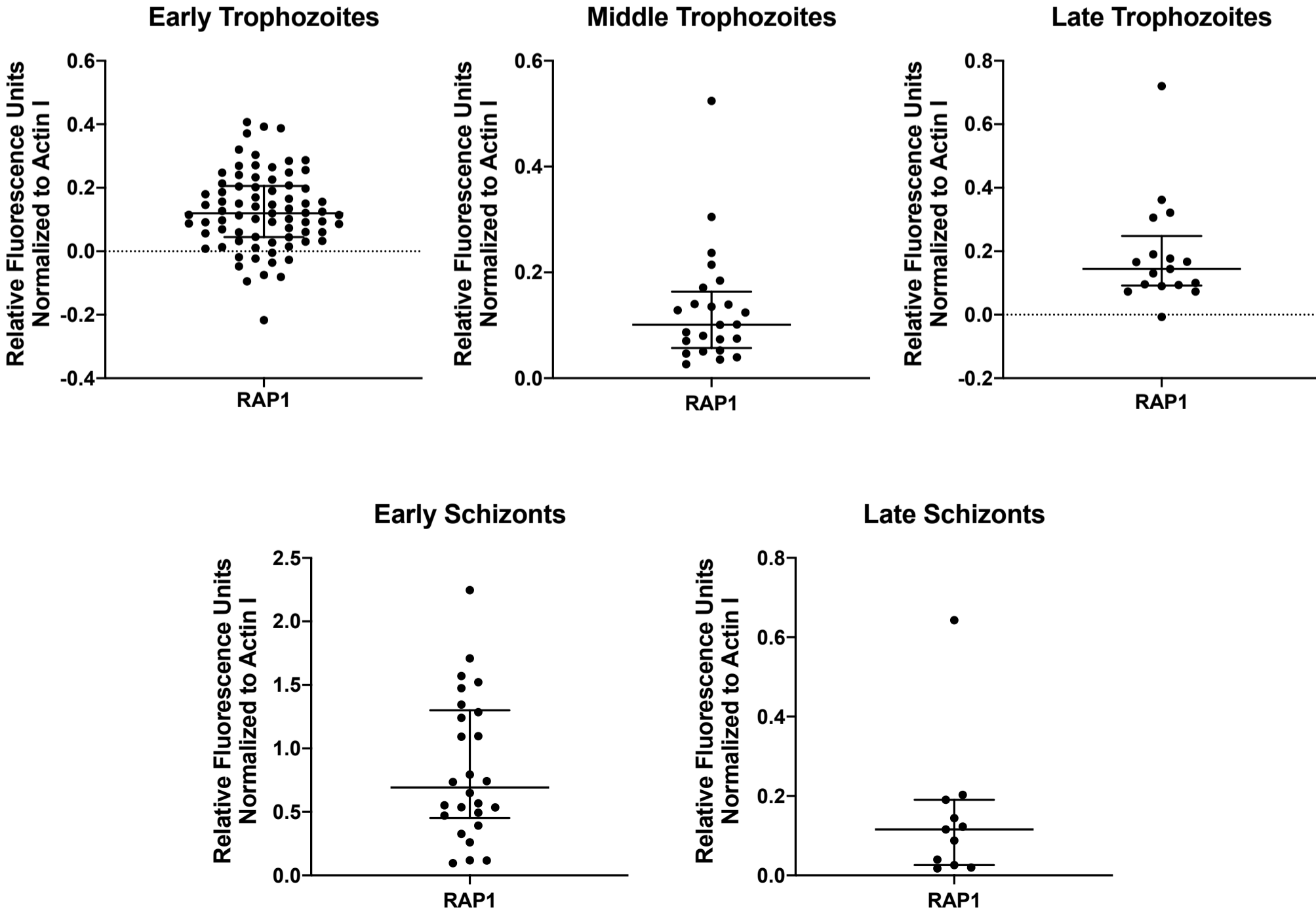

Supplement: S7 Fig — SERA4 shows highest expression in late trophozoites, while RAP1 shows highest expression in early schizonts. Error bars represent the median with the interquartile range. (PDF) [file pgen.1008506.s007.pdf]
