# Supplementary material for: Latent transcriptional variations of individual Plasmodium falciparum uncovered by single-cell RNA-seq and fluorescence imaging
Source: PLoS Genet. 2019 Dec 19;15(12):e1008506. doi: 10.1371/journal.pgen.1008506 (PMC6952112; doi:10.1371/journal.pgen.1008506)

RNA-FISH Signal Intensities of Representative Markers

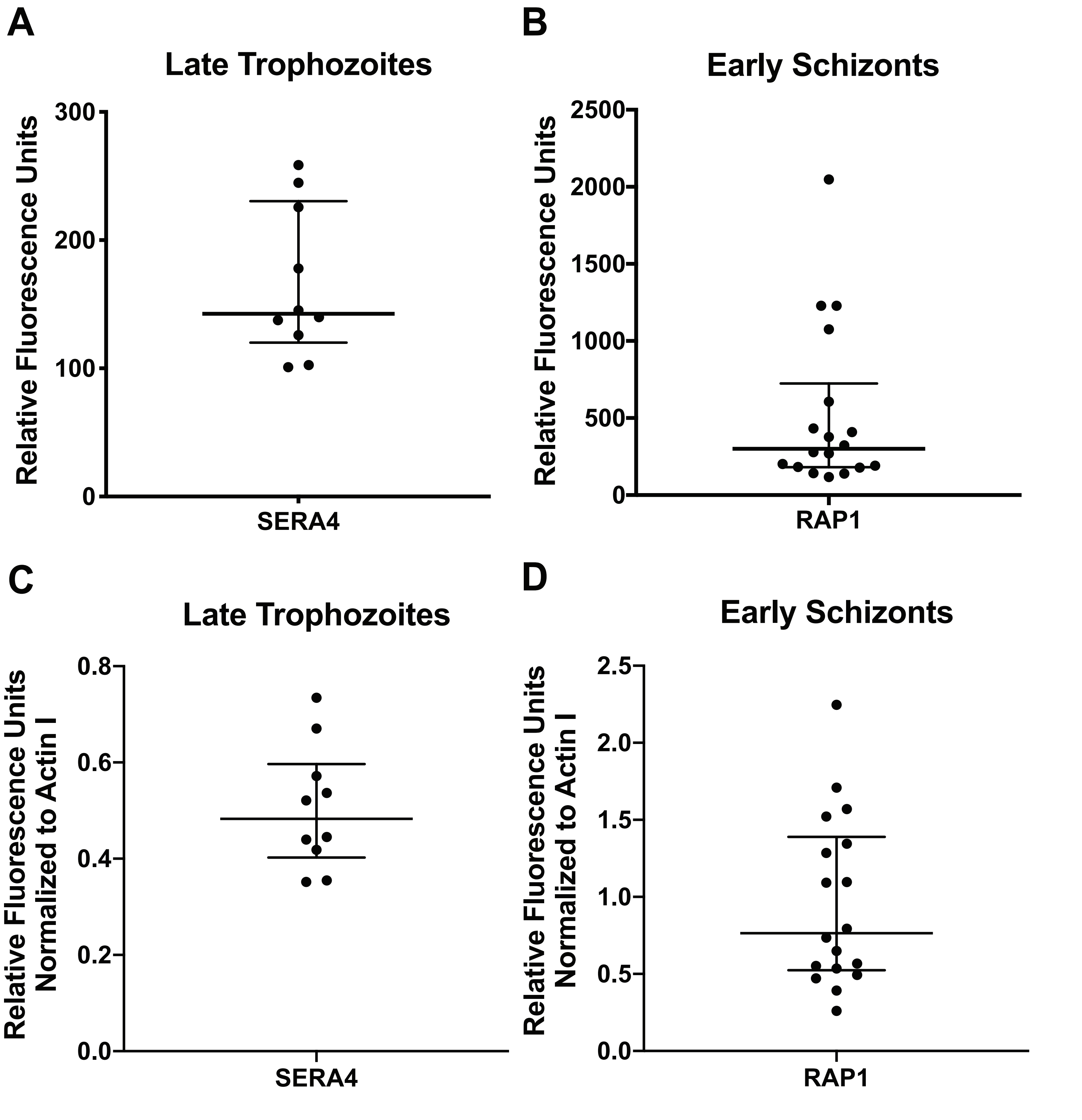

Supplement: S6 Fig — Individual parasites were measured for their mean fluorescence intensity representative of RNA expression for each marker, which showed wide variation in the expression levels among the exclusive marker-expressing parasites. These values were normalized to actin I for C) SERA4 and D) RAP1. Error bars represent the median with the interquartile range. (PDF) [file pgen.1008506.s006.pdf]
