# Supplementary material for: Latent transcriptional variations of individual Plasmodium falciparum uncovered by single-cell RNA-seq and fluorescence imaging
Source: PLoS Genet. 2019 Dec 19;15(12):e1008506. doi: 10.1371/journal.pgen.1008506 (PMC6952112; doi:10.1371/journal.pgen.1008506)
Supplement: S8 Fig — A silhouette width value ranges from -1 to +1, with a high value indicating that the cell is similar to its own cluster but not to its neighboring clusters. (PDF) [file pgen.1008506.s008.pdf]

A

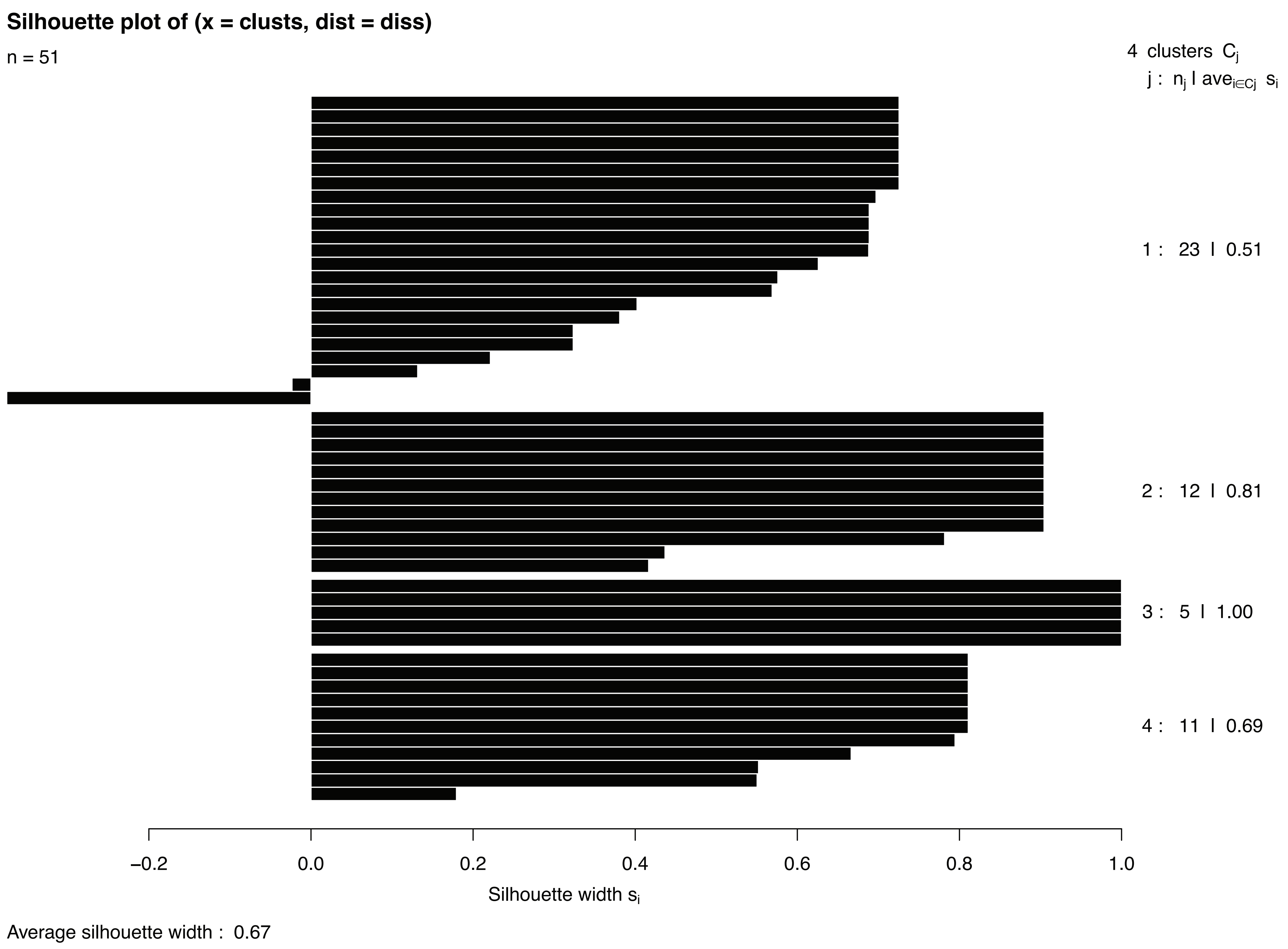

Silhouette plot of (x = clusts, dist = diss)
n = 51
4 clusters C_j
j : n_j | $ave_{i\in Cj}$ s_i
1 : 23 | 0.51
2 : 12 | 0.81
3 : 5 | 1.00
4 : 11 | 0.69
−0.2 0.0 0.2 0.4 0.6 0.8 1.0
Silhouette width s_i
Average silhouette width : 0.67

B

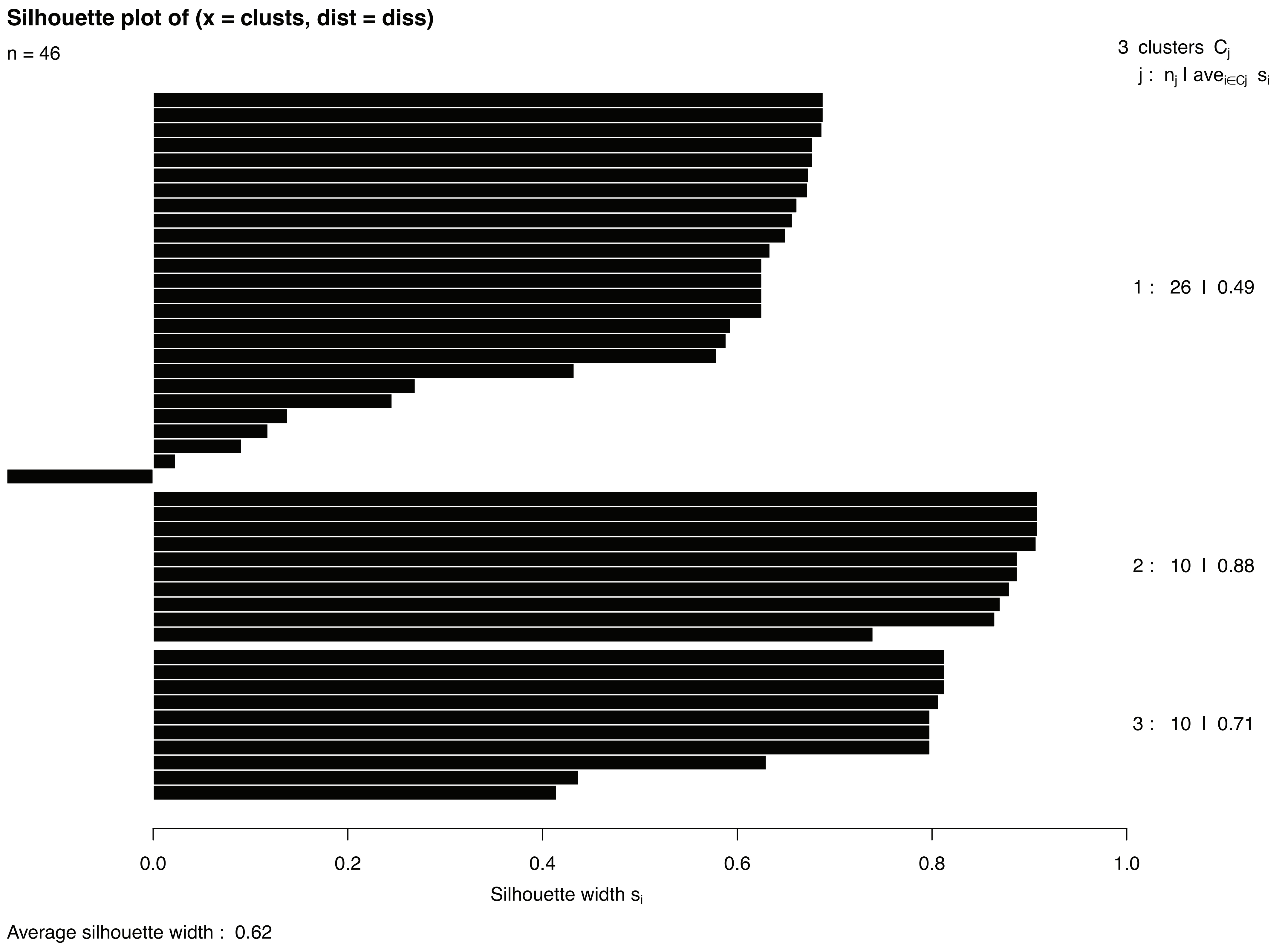

Silhouette plot of (x = clusts, dist = diss)
n = 46
3 clusters C_j
j : n_j | $ave_{i\in Cj}$ s_i
1 : 26 | 0.49
2 : 10 | 0.88
3 : 10 | 0.71
0.0 0.2 0.4 0.6 0.8 1.0
Silhouette width s_i
Average silhouette width : 0.62

C

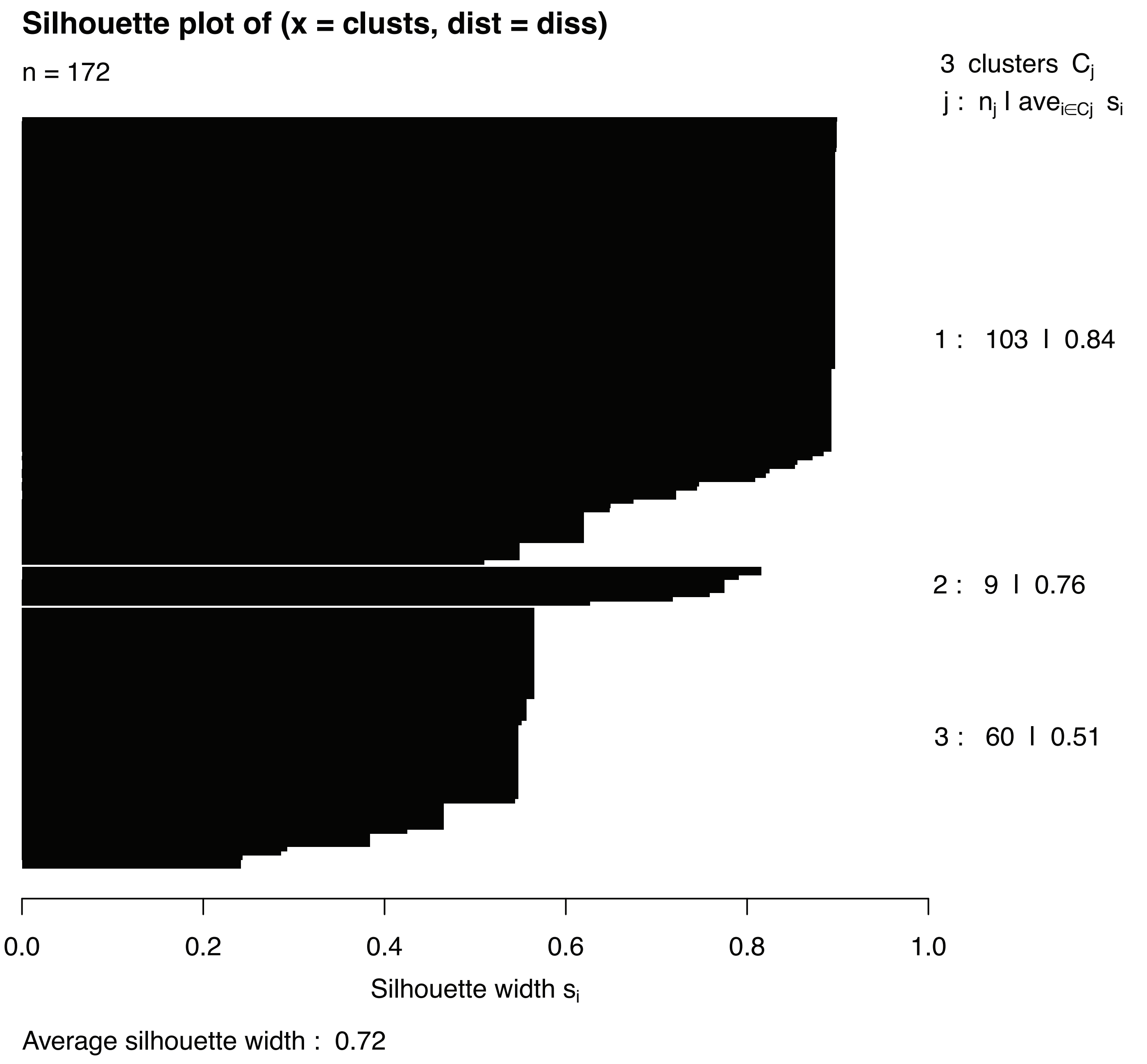

Silhouette plot of (x = clusts, dist = diss)
n = 172
3 clusters C_j
j : n_j | $ave_{i\in Cj}$ s_i
1 : 103 | 0.84
2 : 9 | 0.76
3 : 60 | 0.51
0.0 0.2 0.4 0.6 0.8 1.0
Silhouette width s_i
Average silhouette width : 0.72
